# Structural Remodeling, Redox Regulation, and Metabolic Responses in Cold Plasma Pretreatment-Assisted Drying of Foods: A Critical Review

**DOI:** 10.3390/foods15162887

**Published:** 2026-08-18

**Authors:** Kai Zhang, Qingqing Yuan, Tianrui Liu, Lilang Li, Zhou He, Jianyong Shi, Roujia Zhang, Siyao Liu, Yu Wang, Chenguang Zhou

**Affiliations:** 1 China Light Industry Key Laboratory of Food Intelligent Detection & Processing, School of Food and Biological Engineering, Jiangsu University, Zhenjiang 212013, China; 15936056007@163.com (K.Z.); 19708897519@163.com (Q.Y.); 19709647735@163.com (T.L.); 1000005191@ujs.edu.cn (R.Z.); 2 State Key Laboratory of Discovery and Utilization of Functional Components in Traditional Chinese Medicine, Natural Products Research Center of Guizhou Province, Guizhou Medical University, Guiyang 550014, China; 18336131583@163.com; 3 Cambrian Technology (Suzhou) Co., Ltd., Suzhou 215101, China; algernon@cption.com (Z.H.); jon@cption.com (J.S.); 4 School of Pharmacy, Jiangsu University, Zhenjiang 212013, China; siyaoliu@ujs.edu.cn

**Keywords:** cold plasma, food drying, reactive oxygen and nitrogen species, structural remodeling, moisture migration, redox regulation, metabolic responses

## Abstract

Drying is widely used to stabilize foods, but long processing times and thermal exposure increase energy demand and can impair color, texture, nutrients, and flavor. Cold plasma (CP) pretreatment has attracted interest as a nonthermal strategy for accelerating moisture removal while maintaining product quality. This critical review examines CP pretreatment-assisted drying across food materials by linking structural remodeling with redox regulation and metabolic responses. Current evidence shows that changes in surface wettability, cuticular barriers, cell-wall and membrane integrity, and pore connectivity can facilitate water migration and shorten drying. CP-associated modulation of browning enzymes, oxidative status, and bioactive or flavor-related metabolites may also influence color, antioxidant capacity, nutrient retention, and flavor. However, these effects vary with discharge mode, treatment intensity, gas composition, pressure, temperature, and food-matrix properties. Excessive exposure can instead aggravate oxidation and diminish product quality. Current mechanistic evidence is strongest for plant foods and edible fungi and remains limited for animal-source foods. Together, these findings link plasma-generated chemical and physical agents to structural, biochemical, and drying responses. They provide a basis for defining material-specific operating windows and developing reproducible, safe, and scalable CP pretreatment-assisted drying of foods.

## 1. Introduction

Foods selected for dehydration span fruits, vegetables, grains, seeds, edible fungi, spices, noodles, and processing by-products. Many plant-derived matrices are important sources of vitamins, polyphenols, flavonoids, carotenoids, and volatile flavor compounds [1,2,3]. Fresh fruits and vegetables are particularly susceptible to microbial contamination, tissue softening, and postharvest quality loss because of their high moisture content, active respiration, and fragile cellular architecture [4,5]. Efficient preservation is therefore important for extending shelf life, increasing product value, and reducing losses [6,7].

Drying is a widely used food-preservation operation, particularly for moisture-rich plant materials [8], because lowering moisture content and water activity can slow microbial growth and deterioration and reduce storage and transport burdens. Hot-air drying (HAD) remains common because of its simple equipment and broad material adaptability [9,10]. Nevertheless, heat transfer and internal moisture diffusion can be limited by surface hardening and a prolonged falling-rate period [11,12]. Extended heating may also cause shrinkage, case hardening, browning, loss of thermolabile nutrients, and aroma deterioration [13]. The main challenge is to reduce mass-transfer resistance without introducing unacceptable thermal, oxidative, textural, or sensory damage. Improving drying efficiency can additionally support lower energy use and environmental burdens [14].

Physical-field-assisted pretreatments can alter food structure and moisture-migration pathways before drying. Ultrasound, microwave, pulsed electric field, and CP have all been explored for this purpose [15,16,17]. Such treatments may shorten drying by changing permeability, structure, or water mobility while limiting prolonged heat exposure [8,18]. CP is a partially ionized, thermally nonequilibrium gas containing energetic electrons, ions, excited species, radicals, photons, and electric fields; its classification as nonthermal does not remove the need to measure gas and sample temperatures [19]. RONS generated in or transported from a discharge may modify surface wettability and cellular interfaces, thereby affecting moisture transport and quality formation [20,21]. However, the active agents and delivered dose cannot be inferred from the working gas or applied voltage alone.

Studies in different food matrices report CP-associated changes in drying rate, color, texture, nutrients, and flavor [20,22]. Earlier work, however, has often emphasized parameter optimization or isolated quality indices, while the causal links among discharge physics, species delivery, structure, water state, redox chemistry, metabolism, and drying remain incompletely integrated [23,24]. No universal optimum can be inferred because response depends on discharge mode, absorbed energy, working gas and humidity, pressure, exposure geometry, RONS dose, sample size, matrix structure, and composition [24]. Conditions that improve transport in one matrix can intensify oxidation, pigment loss, cellular damage, nutrient degradation, or off-flavor in another [23]. Broader CP food-safety evidence likewise shows that species profiles, treatment mode, and sample surface properties govern efficacy and boundaries [25]. CP pretreatment-assisted drying is therefore a condition-dependent intervention whose effects are governed by the delivered exposure and food matrix, rather than an intrinsically beneficial intensification step [26,27].

Previous reviews have examined nonthermal drying pretreatments and CP in food processing, including drying performance, proposed mechanisms, energy use, product quality, and industrial prospects [20,22,24]. Du et al. further provided a CP-specific systematic synthesis [28]. Related reviews have addressed multi-technology treatments for fruits and vegetables [29], protein-rich foods [30], and fresh-cut systems [31]. More recent work has synthesized nonthermal drying pretreatments [32] and quantified their effects across fruits and vegetables [33]. These reviews establish that CP can alter drying and product quality, yet the causal sequence from discharge to food response remains incompletely integrated. A food-wide explanation is still needed for how delivered chemical and physical agents reshape surfaces, tissues, and water transport and subsequently interact with redox and metabolic processes. Here, the evidence is organized along the sequence of exposure, structural remodeling, moisture migration, redox regulation, metabolic response, and final drying outcomes. The discussion begins by comparing discharge modes and exposure configurations and then traces how CP-induced structural remodeling affects moisture migration and drying kinetics. Redox and metabolic pathways that shape color, texture, nutrients, and flavor are then examined before these responses are integrated into material-specific process-control and scale-up strategies. Although the scope encompasses foods, current mechanistic evidence is dominated by plant-derived matrices and edible fungi. Accordingly, animal-source foods are discussed without applying plant cell-wall mechanisms. Direct, indirect, hybrid, and EHD-analog studies are discussed separately to compare their differing combinations of chemical and physical action [23,26]. Table 1 compares the scope and thematic focus of eight relevant reviews and the present review.

To define the evidence base, the Web of Science Core Collection was searched from inception to 10 August 2026. Search terms covered cold plasma, cold atmospheric or low-temperature plasma, dielectric-barrier discharge, plasma jets, gliding arc plasma, plasma-processed air, and plasma-activated water, combined with drying, dehydration, or dewatering. The exact search strategy, screening summary, and eligibility criteria are provided in Supplementary Table S1. The search returned 1711 records; 1695 English articles or reviews were screened, and 255 underwent detailed eligibility assessment. Screening retained 85 primary studies (Table S2) that applied a CP-related intervention before or during drying of an edible material and reported at least one drying, moisture transfer, energy, or dried product quality outcome; 8 reviews were used to position the field.

## 2. Cold Plasma Generation Modes and Discharge Systems

**Figure 1:**
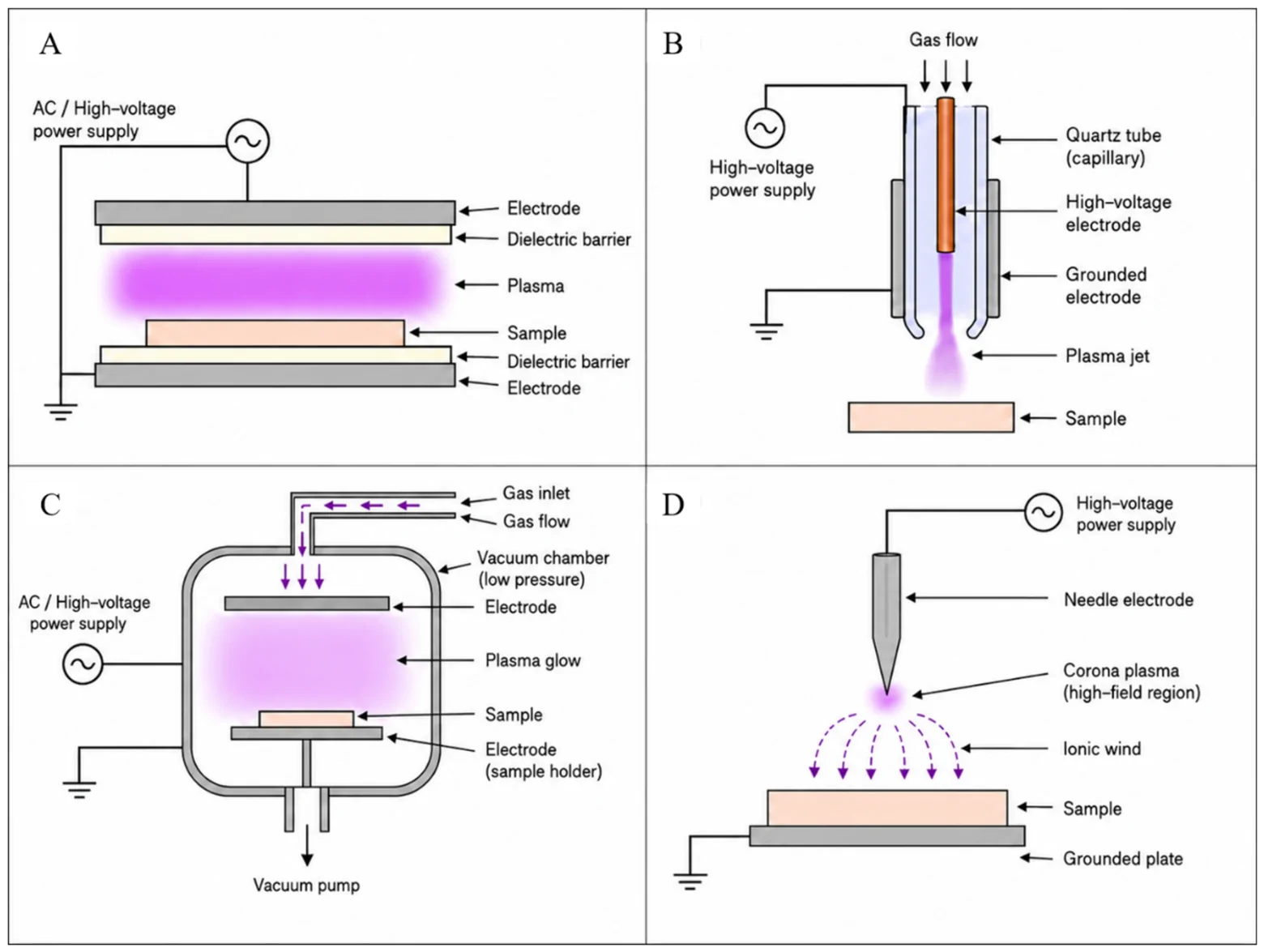
Schematic diagram of typical cold plasma generation modes and discharge types used in food pretreatment: (**A**) dielectric barrier discharge (DBD), (**B**) atmospheric-pressure cold plasma jet (CPJ), (**C**) low-pressure glow discharge (LPGD), and (**D**) corona discharge.

CP is a thermally nonequilibrium, partially ionized gas in which energetic electrons sustain excitation, dissociation, and ionization while the bulk gas and heavy species can remain much cooler [34]. Under typical food-processing conditions, gas and product temperatures generally remain near ambient levels, commonly within approximately 20–50 °C, although higher local temperatures may occur depending on the discharge configuration and operating conditions. Gas temperature and sample surface and core temperatures can therefore diverge and should not be treated as interchangeable. Electrons, ions, electric fields, and ultraviolet radiation can act directly on samples located within the discharge region, whereas remote or afterglow treatments progressively favor the action of transported metastable and longer-lived reactive species. The relative contributions of these agents depend on electrode geometry, applied waveform, gas composition and humidity, pressure, ambient-gas entrainment, treatment distance, and residence time. The principal discharge configurations considered in this review are illustrated in Figure 1.

### 2.1. Dielectric Barrier Discharge (DBD)

Dielectric barrier discharge (DBD) is widely used in food-related CP studies [20]. At least one dielectric separates the electrodes, and alternating or pulsed high voltage produces transient microdischarges while limiting the current and suppressing transition to an arc. A food placed within the active gap can be exposed to charged particles, photons, fields, and short- and long-lived species; a food treated outside the active region receives an afterglow whose composition changes during transport. The two geometries deliver different exposures. The electrode–dielectric configuration is illustrated in Figure 1A.

DBD can provide a comparatively large treatment area and may be integrated into batch or continuous processing, but spatial uniformity depends on electrode shape, gap, dielectric properties, sample loading, and local humidity. RONS and electric-field effects may modify surface chemistry and permeability [35]; however, a visually uniform device does not ensure a uniform delivered dose. For food-drying pretreatment, its performance is governed by discharge uniformity, temperature, and exposure per unit area or mass.

DBD response is influenced by voltage, current, waveform, frequency or duty cycle, treatment time, gas composition and flow, humidity, electrode configuration, and sample position [36]. Electrode geometry also governs microdischarge distribution and species transport [37]. Voltage and current waveforms, real power, frequency and duty cycle, electrode gap and dielectric, gas composition and humidity, treatment geometry, temperature, and treated area or mass collectively determine reactor output. Comparisons based only on nominal voltage or power therefore obscure differences in delivered energy and species transport. Frequency- and configuration-dependent changes reported in apple products illustrate this limitation [38,39].

### 2.2. Atmospheric-Pressure Cold Plasma Jet (CPJ)

An atmospheric-pressure cold plasma jet (CPJ) generates an active discharge in or near a nozzle and transports the plume toward the food. The sample may be exposed directly to the plume or remotely to its afterglow. Carrier gas, flow, humidity, air entrainment, nozzle geometry, treatment distance, waveform, and absorbed power jointly determine the arriving species and thermal load. Consequently, argon and helium jets operated at the same applied voltage cannot be assumed to have the same chemistry, current density, or sample temperature. The remote-treatment configuration is illustrated in Figure 1B.

CPJ treatment can be spatially targeted and can alter surface wettability or morphology, with potential consequences for moisture transport. White grape experiments reported surface activation and faster subsequent drying [40], and shiitake mushroom work extended application to a porous food matrix [41]. These studies demonstrate material-specific performance, although they do not identify which species, charged particles, ultraviolet component, or gas-flow effect caused the response.

A single nozzle treats a limited area, and scaling generally requires scanning or a multi-nozzle array. Species attenuation, air entrainment, gas-flow-induced convection, and between-nozzle variation can then change both dose and drying response. At scale, CPJ exposure is governed by spatial variation in electrical power, gas and sample temperatures, reactive species, and treatment uniformity rather than by nozzle count alone.

### 2.3. Low-Pressure Glow Discharge (LPGD)

Low-pressure glow discharge (LPGD) is generated in a chamber under reduced pressure. The longer electron mean free path can support a stable nonequilibrium discharge, but evacuation is also a process intervention: pressure reduction and gas flow can remove moisture, cool or deform tissue, and change mass transfer even without plasma ignition. These effects can overlap with those of the discharge when pressure history, residence time, gas flow, and sample loading differ between treatments. The chamber and vacuum-pump arrangement is shown in Figure 1C.

Low-pressure treatment can provide broad surface exposure, but plasma and vacuum effects must be separated. In mango slices, oxygen CP at 1.4 mbar and 90 kV was associated with cultivar-dependent drying and biochemical responses [42]. Because the study compared untreated and sodium-metabisulfite-treated material but did not report a matched vacuum-only arm, the independent contribution of reduced pressure cannot be fully resolved. The observed treatment contrast remains valid, but the specific role of plasma cannot be separated from the influence of reduced pressure.

LPGD requires a sealed chamber and vacuum system, increasing equipment and operating requirements [43]. Continuous implementation also depends on load-lock design, pumping capacity, product residence time, and pressure recovery. Mechanistic interpretation and scale-up therefore depend on pressure history, gas throughput, temperature, and the contribution of vacuum alone.

### 2.4. Corona Discharge

Corona discharge arises around a high-field electrode at atmospheric pressure and may generate ions, reactive species, and ionic wind [44]. In food drying, however, many needle-to-plate high-voltage systems are designed primarily as electrohydrodynamic (EHD) dryers. Electrical excitation alone is not sufficient to classify such a study as CP treatment; evidence of discharge and/or chemical exposure is required. The asymmetric geometry and ionic-wind pathway are shown in Figure 1D.

Ionic wind can thin the external boundary layer and increase the convective mass-transfer coefficient, thereby accelerating low-temperature drying [44]. When RONS are not measured and the design lacks a control that separates airflow, electric-field, and discharge chemistry, the result supports an EHD mechanism but not a RONS-mediated CP mechanism. Accordingly, these studies are discussed as EHD analogs that clarify the possible contribution of ionic wind.

Corona systems may also form ozone and nitrogen oxides, but their concentrations depend on polarity, current, electrode geometry, gas composition, and humidity [44]. Accordingly, tissue remodeling cannot be assigned to RONS from the presence of high voltage alone. For powders and other low-moisture foods, sample stacking, shielding, and uneven exposure further complicate interpretation [45].

The four configurations differ in exposure location, charged-particle and photon access, species lifetime, pressure, gas-flow effects, and energy coupling [46]. DBD and LPGD can treat broader areas, CPJ enables localized or remote delivery, and EHD/corona systems may predominantly alter external convection [47]. These differences make the intended mode of action central to device selection and interpretation. The studies summarized in Table 2 and Table 3 were selected to capture the diversity of exposure configurations, food matrices, drying methods, and treatment responses reported in the literature. Table 2 relates reactor configuration and operating conditions to the food application, reported plasma chemistry, and practical implications of each system.

## 3. Structural Remodeling and Moisture-Migration Mechanisms in CP Pretreatment-Assisted Drying

**Figure 2:**
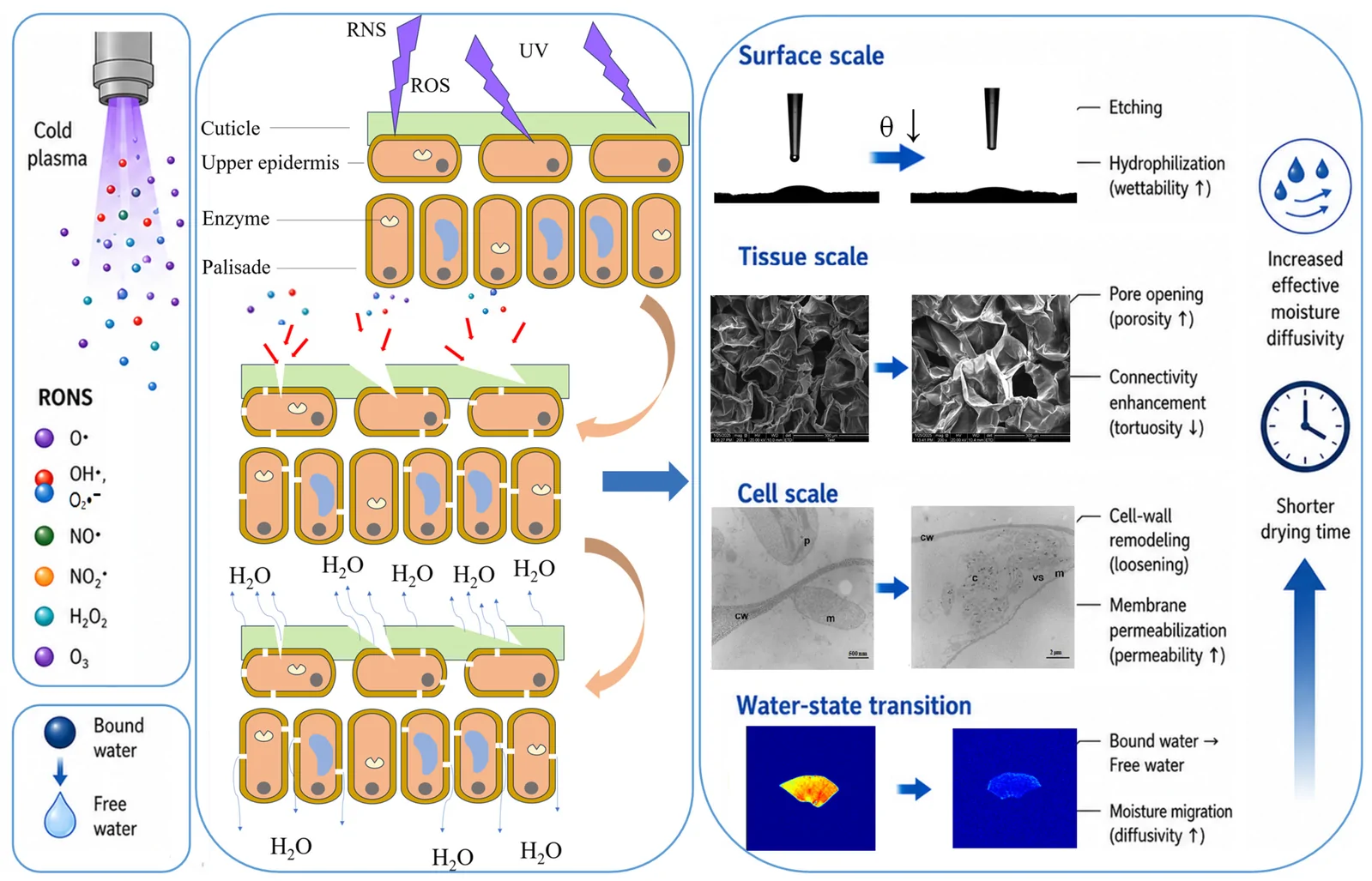
Schematic diagram illustrating the multiscale mass-transfer mechanisms by which cold plasma enhances food drying kinetics, including surface modification, tissue-scale pore opening, cell-scale remodeling, and water-state transition [58,59]. Some panels were reprinted or adapted from Ref. [58] with permission. The TEM micrographs showing tissue ultrastructure were adapted from Figure 5A2 and Figure 5E4 of Ref. [59] under the terms of the Creative Commons Attribution License (CC BY 4.0). Cropping, relabeling, and layout adjustments were performed for consistency.

Across white grape and shiitake mushroom, CPJ pretreatment has been associated with faster dehydration and altered moisture transport [40,41], while low-pressure plasma has improved drying-rate parameters in corn [52]. These material-specific findings converge on a multiscale mechanism in which surface activation, pore-network remodeling, cell-wall or membrane changes, and water-state redistribution reduce external or internal mass-transfer resistance. Figure 2 illustrates the proposed sequence from surface modification and tissue/cellular remodeling to water-state transition, enhanced moisture migration, and shorter drying.

### 3.1. Surface Modification and Tissue Structure Remodeling

For direct exposure, initial plasma-material interactions are expected to be concentrated at the surface, while internal remodeling is supported only when structural changes extend below the exposed surface [35]. RONS, charged particles, and ultraviolet photons can contact the exposed surface, but their relative contributions and penetration depend on reactor geometry and treatment mode. Candidate surface reactions include oxidation of lipids, waxes, and cell-wall components on plant-derived food surfaces, leading to molecular rearrangement and moderate etching [60]. In CP-treated jackfruit slices, surface-related structural changes were associated with altered drying behavior and bioactive-compound retention [54]. Partial oxidation or degradation of hydrophobic compounds exposes additional active sites and creates microscale pathways favorable for water transport. CP may also introduce oxygen-containing polar groups, such as hydroxyl (-OH), carbonyl (C=O), and carboxyl (-COOH) groups, into cell-wall polysaccharides, pectin, and surface organic constituents, thereby increasing surface free energy and reducing the contact angle [46,61]. Improved wettability facilitates water supply to the evaporation interface, alleviates local mass-transfer resistance at the early drying stage, and promotes continuous moisture migration from the interior to the surface. This surface-scale pathway, including etching, hydrophilization, and early moisture-interface regulation, is further illustrated in Figure 2.

In addition to surface modification, CP can induce limited internal tissue remodeling [61]. Plant tissue is a complex porous system composed of cells, intercellular spaces, and the cell-wall matrix, and its internal mass-transfer efficiency is strongly governed by pore structure and pore connectivity. Zhou et al. [49] observed the formation of more micropores on CP-treated goji berries and linked these changes to improved moisture migration and evaporation pathways. Bao et al. [50] also reported CP-induced surface-structure changes in jujube slices and associated them with accelerated drying and quality preservation. In sliced materials, Subrahmanyam et al. [62] further demonstrated that CP pretreatment altered surface and internal structures of apple slices during refractance-window drying, thereby affecting drying kinetics and quality attributes. These findings indicate that pore opening, crack formation, and porous-network remodeling are important structural bases for reduced internal diffusion resistance. However, an increase in pore number alone does not necessarily indicate improved mass transfer; pore connectivity, pore-size distribution, and network integrity are more informative indicators of CP-induced drying enhancement. Chen et al. [63] also showed that CP treatment could affect water-molecule migration and quality changes in fresh wet noodles, suggesting that CP-regulated moisture transport is not limited to fruit and vegetable matrices.

Responses to CP-induced surface modification differ among food matrices [24]. Intact plant foods with cuticular or waxy barriers are more likely to show surface etching and hydrophilization, whereas cut or peeled tissues can show cell-wall and pore-network changes [64,65]. Geometry, tissue compactness, composition, and initial water state also govern species transport and structural response. From an engineering perspective, the key issue is whether a measured exposure lowers mass-transfer resistance without causing structural failure. Reported changes in cell-wall polysaccharides and phytochemical distribution in okra further illustrate this material dependence [61].

### 3.2. Cell-Level Regulation and Moisture-Migration Enhancement

Cell-wall or membrane changes provide a plausible route by which CP exposure could alter moisture release, but this link has rarely been measured directly in the same drying experiment [60]. Cell walls and membranes are important barriers limiting the diffusion of intracellular water to the outside [66]. Their structural state determines the difficulty of water migration from vacuoles and cytoplasm to intercellular spaces, pore networks, and the evaporation surface. Plant cell walls are mainly composed of pectin, cellulose, and hemicellulose, and their three-dimensional network provides mechanical strength while restricting internal moisture diffusion [61]. One proposed mechanism is limited oxidation of cell-wall polysaccharides, resulting in pectin de-esterification, limited polysaccharide-chain scission, and loosening of the middle lamella, thereby decreasing intercellular adhesion and reducing the density of the cell-wall network [36]. Membrane disruption may also alter permeability, facilitating the release of intracellular water into extracellular spaces [35].

Cell-wall remodeling and membrane permeabilization jointly alter water states within tissues [28]. Water in plant tissues is commonly categorized into free water, immobilized water, and bound water, with the latter two forms contributing substantially to internal mass-transfer resistance [63]. As cellular constraints are weakened, part of the immobilized water may shift toward more mobile water states and enter intercellular spaces and connected pore channels, increasing the fraction and mobility of transportable water. Low-field nuclear magnetic resonance (LF-NMR) and magnetic resonance imaging (MRI) can provide evidence for water-state transition, but their direct application in CP pretreatment-assisted drying of foods remains limited. Water-state changes thus provide a plausible intermediate link between structural remodeling and mass-transfer enhancement, but this link requires further validation by multiscale characterization. The cell-scale permeability changes and water-state transition pathway are further illustrated in Figure 2.

Cell-level response is constrained by discharge conditions and tissue tolerance [46]. Limited cell-wall modification and membrane permeabilization may facilitate water release, whereas loss of membrane integrity, leakage, weakened intercellular support, or tissue collapse can impair texture and nutrient retention [60,67]. Concurrent structural changes and water-state shifts under matched treatment conditions provide stronger support for a CP-related increase in moisture migration; device-level guidance for fruit applications likewise emphasizes the interaction between surface properties and treatment intensity [68].

### 3.3. Drying-Kinetics Improvement and Energy Performance

Surface modification, microstructural remodeling, cell-level changes, and water-state transition together provide a plausible link to macroscopic drying behavior, although few studies have measured every step within one experiment [69]. Where these changes are demonstrated, they may reduce internal or external mass-transfer resistance and shift drying curves toward faster dehydration. In white grape, CPJ-induced surface activation was linked with faster water removal [40], while shiitake mushroom studies associated CP pretreatment with surface morphology changes and microchannel formation during hot-air drying [41]. Corn-kernel and jackfruit studies reported microstructural or diffusivity changes alongside faster drying [52,54]. A low-pressure mango study reported material-specific drying responses, although the absence of a vacuum-only control limits plasma-specific attribution [55]. Collectively, these studies are consistent with contributions from surface channels and internal diffusion, although concurrent gas-flow, pressure, and heating effects preclude attribution to a single mechanism [70,71].

Thin-layer drying models, such as the Page, Henderson–Pabis, Midilli–Kucuk, and logarithmic models, are commonly used to quantitatively assess drying enhancement. Effective moisture diffusivity (Deff), drying-rate constant (k), and activation energy (Ea) further reflect mass-transfer characteristics. In corn kernels, low-pressure plasma pretreatment reduced drying time and activation energy [52], whereas jackfruit slices showed CP-dependent changes in drying behavior under different temperatures [54]. Low-pressure CP-treated mango also provides material-specific evidence for improved drying kinetics and moisture migration [55]. Their interpretation, however, needs to account for tissue type, sample geometry, discharge conditions, and drying mode; increasing CP intensity does not produce a universal kinetic response.

Improved drying kinetics can reduce energy use during the drying phase. Energy consumption in conventional hot-air drying is mainly concentrated in the prolonged falling-rate period [72,73]. By enhancing internal diffusion and shortening the time required to reach the target moisture content, CP pretreatment may reduce operating time and thermal load. Gliding-arc plasma studies on grape and saffron illustrate how brief pretreatment can shorten drying and reduce the energy demand associated with the drying operation [74,75]. More recently, improved diffusivity and shorter drying in green pea further indicated how material-specific plasma responses may influence the energy demand of subsequent drying [76]. The response may become non-monotonic as stronger exposure simultaneously increases thermal and oxidative loads. Heating, leakage, or structural damage can then offset gains in mass transfer [60,67]. The overall energy balance, however, depends on the system boundary. Shorter drying lowers the thermal demand of the dryer, whereas net process energy also reflects plasma generation and, where applicable, gas supply, vacuum, cooling, and other auxiliary loads. Because plasma exposure is often brief relative to drying, its contribution may be small in some systems, while auxiliary requirements can alter the balance in others. Separating drying-stage and total-process energy will clarify how reactor design and material-specific kinetic gains translate into overall energy performance.

## 4. Redox and Metabolic Regulation of Product Quality

CP pretreatment can influence color, texture, nutritional value, and flavor by modifying the duration of thermal exposure, tissue integrity, redox chemistry, and metabolism [1]. Structural remodeling and altered moisture transport provide the physical link to drying, whereas enzyme activity, antioxidant buffering, pigment stability, and flavor-precursor chemistry shape quality retention or deterioration [36]. Their interactions across major quality attributes and their dependence on treatment intensity and food matrix are examined below.

### 4.1. Color Preservation and Browning Inhibition

Color is a key sensory attribute affecting consumer acceptance of dried foods, and it also reflects enzymatic browning, nonenzymatic browning, and pigment stability [77,78]. Prolonged hot-air drying typically decreases lightness (L*), increases total color difference (ΔE), and elevates the browning index (BI) [79]. Some studies report color preservation after selected CP pretreatments, but the outcome varies with matrix, exposure, and subsequent drying history. Farias et al. [38] showed that DBD excitation frequency affected enzymatic activity, antioxidant capacity, and phenolic content in apple cubes and juice. A later comparison of two CP systems confirmed that treatment configuration can alter antioxidant and enzyme-related responses [39]. The candidate species proposed in these studies are plasma-derived •OH, O_2_•^−^, HO_2_•, and •NO. Their effects appear matrix- and exposure-dependent: amino acid oxidation and conformational change may suppress PPO or POD, whereas •OH-associated stress may increase POD activity in intact tissue. Resolving the responsible pathway will require concurrent measurement of reactive species and enzyme activity. Oxidative signaling may also alter antioxidant defense systems, including SOD, CAT, and APX, but direct evidence in CP pretreatment-assisted drying remains limited. Zeng et al. [80] supported the substrate-dependent nature of CP-mediated enzyme inactivation using brown rice POD.

CP may also indirectly suppress nonenzymatic browning by improving drying kinetics. By enhancing moisture migration and shortening the overall drying period, CP-treated shiitake mushrooms and jackfruit slices may experience reduced residence time in high-temperature environments [41,54]; green pea studies similarly indicate that improved diffusivity can be accompanied by better color retention [76]. Shorter thermal exposure can decrease the extent of Maillard reaction, caramelization, and ascorbic acid degradation, thereby reducing brown polymer accumulation [81]. Bao et al. [50] reported that suitable CP pretreatment accelerated drying of jujube slices while improving color and antioxidant-related quality, and Zhou et al. [49] observed links among CP treatment, color, rehydration, and effective moisture diffusivity in goji berry. Enzyme-activity assays, total phenolic and pigment analyses, and FTIR measurements provide evidence for CP-regulated color changes, although the direction and magnitude of changes vary with material and treatment intensity. Pigment-focused studies further indicate that CP can affect chlorophylls, carotenoids, anthocyanins, and betalains, implying that color preservation cannot be attributed solely to shortened drying time [82].

Color response depends on the relationship between delivered exposure and the antioxidant-buffering capacity of the matrix [82]. Under suitable conditions, changes in PPO/POD activity can coincide with reduced enzymatic browning [38]; under stronger oxidative conditions, anthocyanins, chlorophylls, carotenoids, and phenolic substrates may instead be degraded [60,83]. Color preservation therefore reflects the combined effects of enzyme activity, pigment stability, antioxidant response, and drying history [84], within matrix-specific limits for color change and oxidation [68]. Broader CP quality reviews similarly describe coordinated enzyme, pigment, and tissue-stress effects [85].

### 4.2. Tissue Structure Preservation and Texture Regulation

Texture is determined by tissue integrity, shrinkage, and rehydration behavior [16,77,86]. Prolonged hot-air drying can promote cell-wall shrinkage, middle-lamella separation, and skeletal collapse [87,88]. When CP shortens drying without undermining structural support, reduced thermal exposure and more uniform water migration may limit irreversible deformation [28,89,90]. This hypothesis is best tested by pairing structural imaging with mechanical measurements, because shorter drying alone does not establish textural preservation.

Preservation of tissue structure further affects mechanical properties and rehydration behavior [91]. CP-pretreated samples have been reported to retain desirable hardness, crispness, and elasticity. Reduced collapse and densification help prevent excessive hardening during conventional hot-air drying, whereas retention of cellular framework and pore structure maintains necessary mechanical strength. Subrahmanyam et al. [62] reported that CP pretreatment improved drying kinetics and quality attributes of apple slices. Goji berry and jujube-slice studies linked CP-induced pore formation and surface modification with rehydration, color, and antioxidant-related quality responses [49,50], while green pea work further associated epidermal micropores and increased surface roughness with improved rehydration and color retention [76]. These CP-focused examples suggest that structural integrity, pore connectivity, and moisture migration jointly influence final texture and rehydration properties.

Texture responses remain matrix- and dose-dependent. Excessive oxidation of wall polysaccharides or disruption of the middle lamella can cause softening, fragmentation, poorer integrity after rehydration, or undesirable mechanical properties [92,93,94]. Cross-study comparison is further shaped by material geometry, wall composition, final moisture basis, water activity, and the selected mechanical endpoint; device-focused reviews likewise emphasize matching treatment conditions to raw-material properties [68].

### 4.3. Preservation of Nutritional and Bioactive Compounds

Bioactive compounds are important indicators of the functional value of dried foods, including vitamin C, anthocyanins, carotenoids, polyphenols, flavonoids, and phenolic acids [1,95]. Thermal exposure and oxidative environments during conventional hot-air drying can degrade thermosensitive compounds and oxidize phenolics [77,96]. Selected CP conditions have been associated with retention of some bioactive compounds, potentially through shortened drying and stress responses; the direction and magnitude depend on material type, exposure, and reactive-species composition [28]. Loureiro et al. [53] showed that CP excitation frequency influenced fruit drying and bioactive-compound retention. Two candidate pathways are reduced thermal loss and altered stress metabolism, but their relative contributions are seldom isolated. Studies on CP processing of fruit juices and fruits also use ascorbic acid, phenolics, and antioxidant activity as indicators of the balance between mild processing and oxidative damage [97].

Plasma-derived RONS can act as both stress signals and oxidants in plant tissues, and the prevailing response depends on species composition and exposure [98]. The CP literature proposes RONS-responsive phenylpropanoid signaling, while related non-CP drying studies provide only an indirect analog for phenylalanine ammonia-lyase-linked secondary-metabolite accumulation [36,99]. At species level, the longer-lived H_2_O_2_ and NO_x_-derived RNS are plausible participants in stress signaling, whereas strongly oxidizing O_3_, •OH, and ^1^O_2_, together with surface-reactive O_2_•−, can promote oxidation of ascorbate, phenolics, and anthocyanins when exposure exceeds antioxidant buffering; carotenoid-specific routes remain unresolved [97,100]. In CP pretreatment-assisted drying, hormesis remains a dose-dependent hypothesis rather than an established outcome [98]. Consistent with this dual response, Lu et al. [101] reported that nitrogen and air plasma shifted Djulis bioactive compounds in opposite directions and attributed the contrast to working-gas-dependent RNS/ROS composition.

HPLC, LC-MS, and metabolomics provide insight into the molecular basis of CP-regulated nutritional quality, as illustrated by integrated metabolomic and transcriptomic analyses of CP-treated broccoli sprouts and postharvest blueberries [102,103]. Current evidence suggests that CP may influence phenylpropanoid metabolism, ascorbate metabolism, and antioxidant-defense networks, thereby modulating the accumulation of polyphenols, flavonoids, and phenolic acids. Nevertheless, explanations of metabolic responses still rely largely on metabolite-profile changes, and causal evidence connecting transcriptional regulation, enzyme activity, and metabolic flux remains insufficient [104]. Although these studies are not all drying-specific, they provide mechanistic references for interpreting CP-induced redox signaling and metabolic responses. Future work should integrate metabolomics, proteomics, and transcriptomics within CP pretreatment-assisted drying systems to establish quantitative links among RONS characteristics, metabolic responses, and nutritional quality formation. Related studies also suggest that plasma-derived systems may alter phenolics, vitamins, and enzyme activity [105].

### 4.4. Flavor Formation and Metabolic Regulation

Flavor reflects volatile compounds, soluble sugars, organic acids, amino acids, and their thermal and oxidative transformations [106,107]. Conventional hot-air drying can remove or transform esters, alcohols, aldehydes, and terpenes [108]. CP-related flavor reviews indicate that lower oxidative exposure may preserve characteristic aromas, whereas stronger oxidation can favor lipid-derived aldehydes or ketones and the loss of esters [109,110,111]. Because drying method itself strongly affects volatile retention [112,113], separating CP-induced flavor changes from drying effects depends on matched drying controls, quantitative volatile profiles, and, ideally, sensory evaluation.

Beyond passive flavor retention, CP may reshape flavor through interactions between oxidative exposure and material-specific precursor pathways. In the PAW-treated rocket series, earlier characterization identified H_2_O_2_, NO_2_^−^, and dissolved O_3_ [105]. A related volatile study reported higher hexan-1-ol and hex-3-en-1-ol and proposed either LOX activation or ROS-promoted linoleic-acid oxidation [109]. Because individual species were not separated, this result supports a pathway-level interpretation rather than a species-specific causal claim. Across other matrices, CP-associated GABA enrichment in germinated grains and non-CP shiitake drying illustrate stress-metabolite accumulation and process-dependent flavor-precursor transformation, respectively [114,115]. LOX-related processes also appear in a CP flavor review and a non-CP postharvest-defense study, although their endpoints and causal contexts differ [109,116]. Non-CP tomato peeling likewise altered LOX activity, carotenoid profiles, and volatile composition, illustrating common processing dependence rather than a CP-specific causal pathway [117]. Together, these findings support a material-specific metabolic interpretation, while direct CP-drying evidence remains limited. The role of individual species remains unresolved until their occurrence and the corresponding pathway response are linked within the same drying experiment.

GC-MS, GC-IMS, and metabolomics are important tools for deciphering CP-regulated flavor formation because they can characterize changes in esters, aldehydes, alcohols, terpenes, and carotenoid-derived volatiles [109]. Existing studies usually interpret precursor transformation through fatty acid, amino acid, sugar, and carotenoid metabolism [118]. However, because flavor-precursor composition varies greatly among materials, CP may lead to flavor retention, remodeling, or deterioration. Direct evidence from VOC omics and sensory evaluation after CP-only food drying remains limited. Studies in spices have shown that CP pretreatment can affect essential oil extraction or volatile compound profiles in cumin and turmeric. These findings provide mechanistic analogies, rather than direct evidence, for flavor changes during fruit and vegetable drying [119,120]. Studies on cinnamon, black pepper, fennel, and black pepper further suggest that microbial control and physicochemical quality changes induced by CP in spices are material-dependent [121].

### 4.5. A Structural–Redox–Metabolic Model of Quality Formation

Building on the earlier CP literature [24], this review integrates the reported quality outcomes into a structural–redox–metabolic model of product quality formation. Within this model, surface hydrophilization, pore opening, and cell-wall or membrane changes may alter mass-transfer resistance and thereby contribute to drying, texture, or rehydration outcomes. Changes in PPO/POD activity and antioxidant defenses, together with possible H_2_O_2_- and NO_x_-derived signaling through phenylpropanoid and ascorbate metabolism, may influence enzymatic browning and pigment protection. By contrast, O_3_, •OH, ^1^O_2_, and O_2_•− can intensify oxidation when matrix buffering is exceeded, shifting the balance toward pigment degradation and oxidative damage [85]. Metabolic responses may contribute to the retention or transformation of polyphenols, flavonoids, phenolic acids, sugars, organic acids, amino acids, and volatile flavor precursors and thus to nutritional and flavor outcomes [36]. The direction of response depends on delivered exposure and matrix buffering; no universal beneficial RONS range has been established, and stronger exposure can introduce oxidative or structural damage [67]. Studies on low-pressure CP treatment of saffron also show that microbial safety, biochemical attributes, and quality preservation require a balance between treatment intensity and product characteristics [122,123].

**Figure 3:**
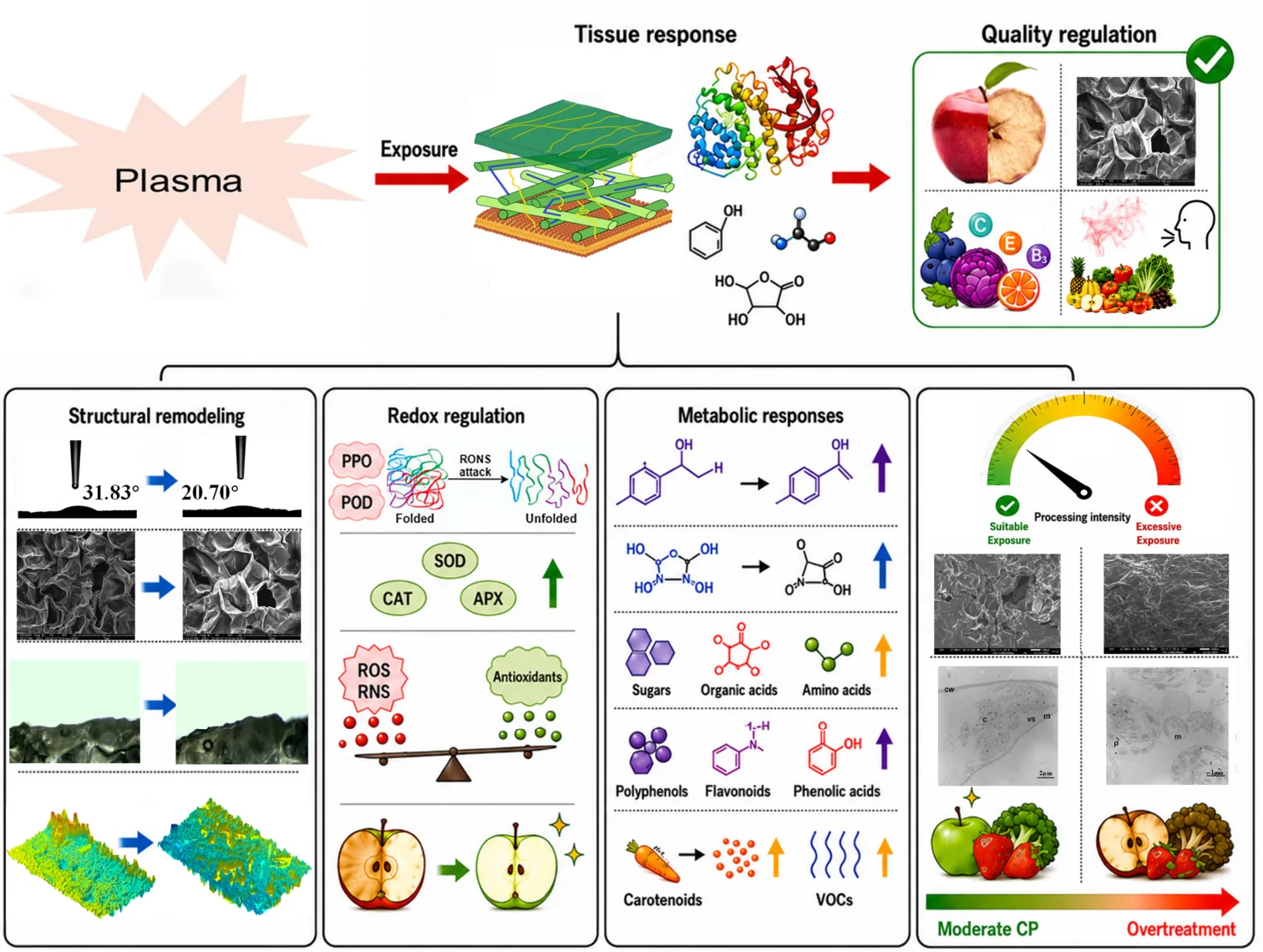
Schematic diagram of the structural–redox–metabolic model for quality regulation and the boundary effects of moderate treatment versus overtreatment in cold plasma pretreatment-assisted drying of foods [28,58,59]. Arrows indicate the direction of treatment-associated transitions or responses, while the green-to-red gradient represents the progression from suitable/moderate CP exposure to excessive exposure and overtreatment. Other colors are used for visual differentiation and do not represent a quantitative scale. Some images/panels were reproduced or adapted from Refs. [28,58] with permission. The TEM micrographs showing ultrastructural changes under moderate plasma-treated water exposure and oxidative-stress-induced disruption were adapted from Figure 5E4 and Figure 5G4 of Ref. [59] under the terms of the Creative Commons Attribution License (CC BY 4.0). Cropping, relabeling, and layout adjustments were performed for consistency.

Overall, structural remodeling, moisture transfer, redox regulation, metabolic response, and quality formation follow an exposure-dependent sequence [94]. The contribution of each link varies among foods. Surface barriers, cellular architecture, composition, antioxidant buffering, and the identities, lifetimes, and reactivities of delivered species, including H_2_O_2_, NO_x_-derived RNS, O_3_, •OH, ^1^O_2_, and O_2_•−, jointly shape exposure and response. Figure 3 summarizes this sequence, and Table 3 brings together a selection of the reported drying, quality, and mechanistic responses.

## 5. Process-Control Strategies for Coordinating Drying Efficiency and Quality

**Figure 4:**
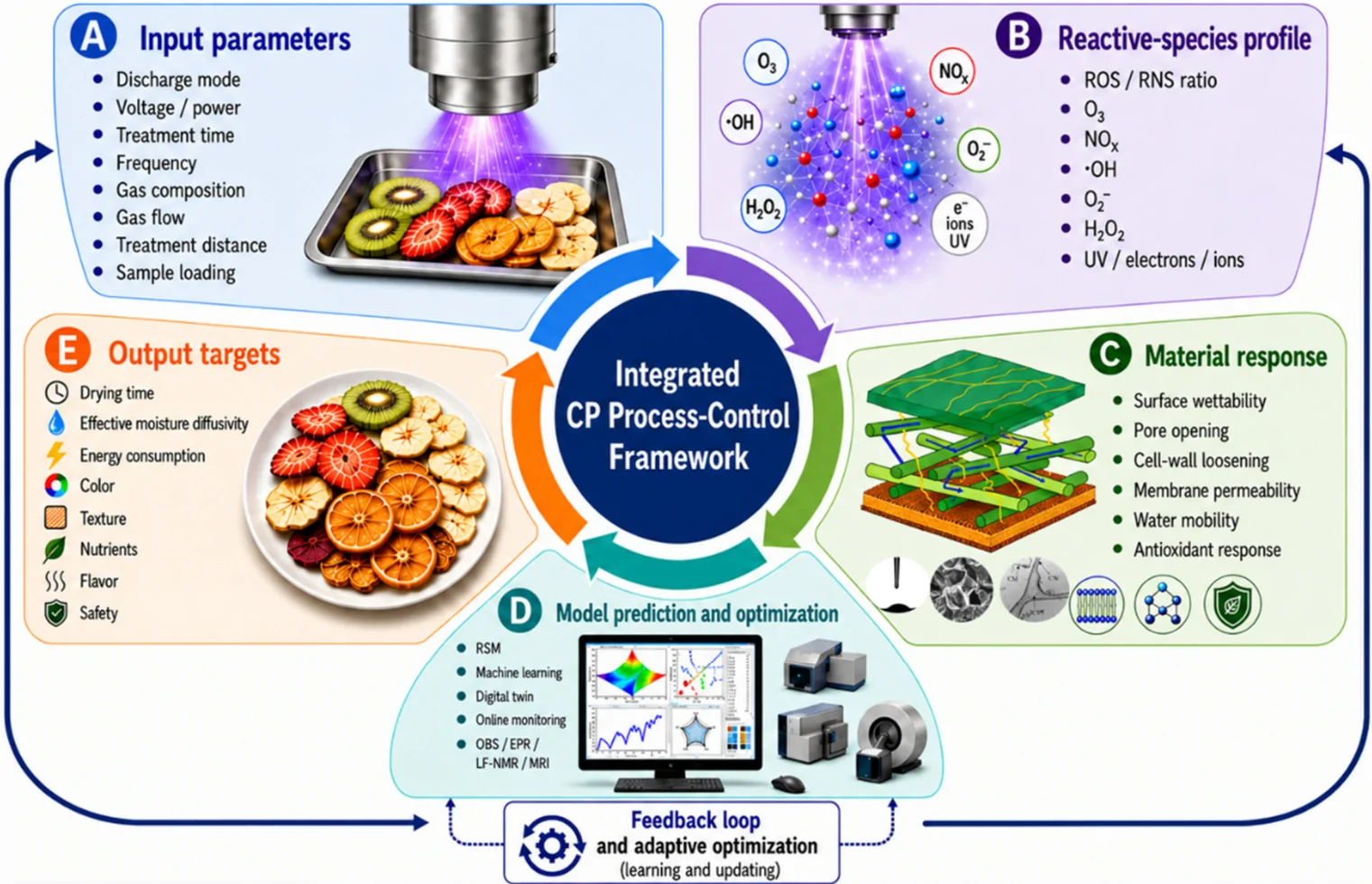
Schematic diagram of an integrated cold plasma (CP) process-control framework for coordinating drying efficiency and product quality in food drying, linking input parameters, reactive-species profiles, material responses, model prediction and optimization, output targets, and feedback-based adaptive optimization.

The performance of CP pretreatment-assisted drying depends on how reactive-species generation is matched to tissue response, mass transfer, and quality formation. A faster drying rate is beneficial only if tissue damage, nutrient degradation, and flavor deterioration are also avoided [20]. Effective CP process optimization balances drying efficiency and product quality by considering discharge-energy input, gas composition, food-matrix properties, and multifactorial interactions. Figure 4 presents this control process as a closed loop in which operating inputs (Figure 4A) determine the reactive-species profile (Figure 4B), trigger material responses (Figure 4C), and are then refined by model prediction, output evaluation, and feedback optimization (Figure 4D,E). In broader drying systems, process optimization should also account for energy efficiency, renewable-energy utilization, and environmental sustainability [14].

### 5.1. Discharge-Energy Input and Processing-Window Optimization

Discharge-energy input can influence plasma chemistry, thermal load, and tissue response, but nominal power or voltage does not directly specify the delivered RONS dose. The input layer in Figure 4A therefore combines discharge mode with voltage or power, exposure time, frequency, gas flow, treatment distance, and sample loading. Low-input settings may produce no detectable response, whereas higher input can alter several chemical and physical exposures at once, with reactor- and matrix-specific effects on species delivery and tissue modification. Drying rate and quality need not improve synchronously as input increases, and exposure-response relationships therefore remain specific to the reactor and food matrix [28]. Loureiro et al. [53] further indicated that excitation frequency and processing conditions influence fruit drying and the retention of bioactive compounds.

CP response can be non-monotonic: increasing input can initially increase surface activation or moisture transfer but can later intensify heating, oxidation, leakage, or structural failure [20,60]. The optimum is an experimentally demonstrated range that satisfies prespecified drying, quality, safety, and energy criteria; it cannot be defined by the largest power or longest exposure.

Cross-study optimization remains difficult because voltage, power, or exposure time alone does not describe the dose delivered to a food [46]. The resulting exposure is shaped by electrical energy, treatment geometry, gas and pressure, thermal history, sample load, and the species generated along the proposed pathway [43]. These variables form the input layer that links reactor operation to material response in Figure 4 and Table 2.

### 5.2. Gas Composition and Reactive-Species Regulation

Gas composition influences discharge behavior and the species that may reach a food surface [47], but a feed-gas label is not a reactive-species measurement. Optical emission spectroscopy can identify emitting excited species in the discharge yet does not, by itself, quantify the chemical dose delivered to a wet or solid sample. Short-lived radicals are better investigated through electron paramagnetic resonance or validated scavenging approaches, while longer-lived compounds such as ozone, hydrogen peroxide, nitrite, and nitrate require calibrated gas-, liquid-, or surface-phase assays. Species profiles are shaped by the sampled phase and position, while their quantitative interpretation depends on concentration or flux and the delay after treatment [101].

Species-specific mechanisms remain hypotheses until they are tested directly. Hydroxyl radicals and atomic oxygen are highly reactive and short-lived, making near-surface oxidation, etching, and biomolecule modification plausible; ozone and hydrogen peroxide can persist longer and contribute to oxidative load, wettability changes, and enzyme modification. Nitric oxide and nitrogen dioxide chemistry, as well as nitrite or nitrate in hydrated matrices, may participate in redox or signaling pathways but can also create residue and sensory concerns. Charged particles and electric fields may alter interfacial permeability, while ultraviolet radiation primarily affects directly exposed regions [35]. These pathways are consistent with reported color, pigment, and flavor sensitivity to oxidative exposure [82,109], but their relative contribution in a given drying experiment remains unresolved unless exposure is linked experimentally to response.

No universally favorable RONS combination or concentration can currently be recommended for food drying. Air- or oxygen-containing systems often favor oxygen chemistry, whereas nitrogen-containing feeds can shift nitrogen pathways [47]; actual output still depends on power coupling, humidity, pressure, and residence time. Argon and helium can stabilize jets, but different metastable energies, air entrainment, current density, and gas heating mean that the carrier-gas name alone cannot predict dose or biological effect. Gas selection is therefore most informative when it is based on measured exposure: a target species profile, quantified delivered dose, and matrix-specific drying, quality, and safety limits [101].

### 5.3. Tissue Characteristics and Process Adaptation

Food matrices differ in geometry, porosity, surface barriers, composition, initial water state, and oxidative buffering, so identical CP settings can produce different drying and quality responses [127,128]. The available mechanistic literature is dominated by fruits, vegetables, grains, seeds, and edible fungi [20]; animal-source evidence is currently too sparse to justify direct transfer of plant cell-wall mechanisms. Consequently, plant-specific mechanisms are not extended to the entire food category.

Within plant-derived foods, porous tissues may respond at lower exposure, whereas dense roots or tubers can retain higher internal diffusion resistance [129,130]. Thin-walled berries may be especially sensitive to leakage or collapse [103,131]. Mango, apple, green pea, and mushroom reports illustrate material-specific responses under distinct experimental conditions [42,62,76,127]. These examples do not establish a universal hierarchy of sensitivity; instead, geometry, composition, water state, and oxidative buffering shape cross-material responses, as summarized in Table 3.

Cell-wall composition, initial moisture content, and raw-material form also affect CP performance. Tissues rich in pectin are more likely to undergo middle-lamella loosening and pore expansion, whereas tissues with higher cellulose or lignin proportions often have greater structural stability [61,132]. When intact fruits have cuticular or waxy layers, CP mainly induces surface etching and hydrophilization; in peeled, sliced, or diced samples, cell-wall remodeling and optimization of internal mass-transfer pathways are more evident [28]. These linked responses, including surface wettability, pore opening, cell-wall loosening, membrane permeability, water mobility, and antioxidant responses, are summarized in Figure 4C. Materials rich in phenolics and antioxidants usually possess stronger oxidative-buffering capacity, whereas materials with weaker antioxidant capacity are more prone to pigment degradation and lipid oxidation [133,134]. These differences favor material-specific process design based on tissue structure, cell-wall composition, water status, and metabolic background. Studies on blueberries and mango cultivars also indicate that tissue physiology, cultivar, and microstructural differences affect CP-induced redox, drying, antioxidant, and microbial responses [42,103].

### 5.4. Multifactorial Optimization and Precision Control

CP pretreatment-assisted drying involves coupled effects of discharge parameters, gas composition, material properties, and drying conditions. Single-factor experiments are therefore insufficient to identify robust process combinations. Multifactorial optimization can instead balance drying efficiency with product quality [24]. RSM is particularly useful for relating treatment variables to drying time, effective moisture diffusivity, energy consumption, color, texture, nutrients, flavor, and safety (Figure 4E). Namjoo et al. [135] applied this approach to optimize caraway-seed drying.

Machine learning (ML) may support optimization of CP pretreatment-assisted drying [136,137]. Compared with traditional statistical models, ML can handle complex nonlinear relationships among discharge parameters, reactive-species characteristics [138], raw-material attributes, and quality responses [139], enabling rapid prediction of drying behavior and product quality [140]. These capabilities correspond to the prediction and optimization stage in Figure 4D. However, applications of ML to CP pretreatment-assisted drying remain limited, and ML currently represents a prospective precision-control tool rather than an established industrial technology [141].

Digital twin technology has emerged in food processing as a platform for monitoring and model-predictive control and may also support CP pretreatment-assisted drying [142,143,144]. By building a virtual model synchronized with the real process, it may simulate discharge behavior, mass transfer, and quality changes in real time, while online sensing and intelligent algorithms enable dynamic parameter control [145,146] (Figure 4D). Direct applications of digital twins in CP pretreatment-assisted drying of foods still require further validation. These developments indicate a shift from experience-driven to data-driven optimization of CP pretreatment-assisted drying. Integrating RSM, ML, digital twins, and online monitoring could link operating conditions and measured exposures with material responses and product targets, supporting material-specific optimization of drying efficiency, energy consumption, and product quality.

## 6. Material-Dependent Mechanisms and Process Translation

**Figure 5:**
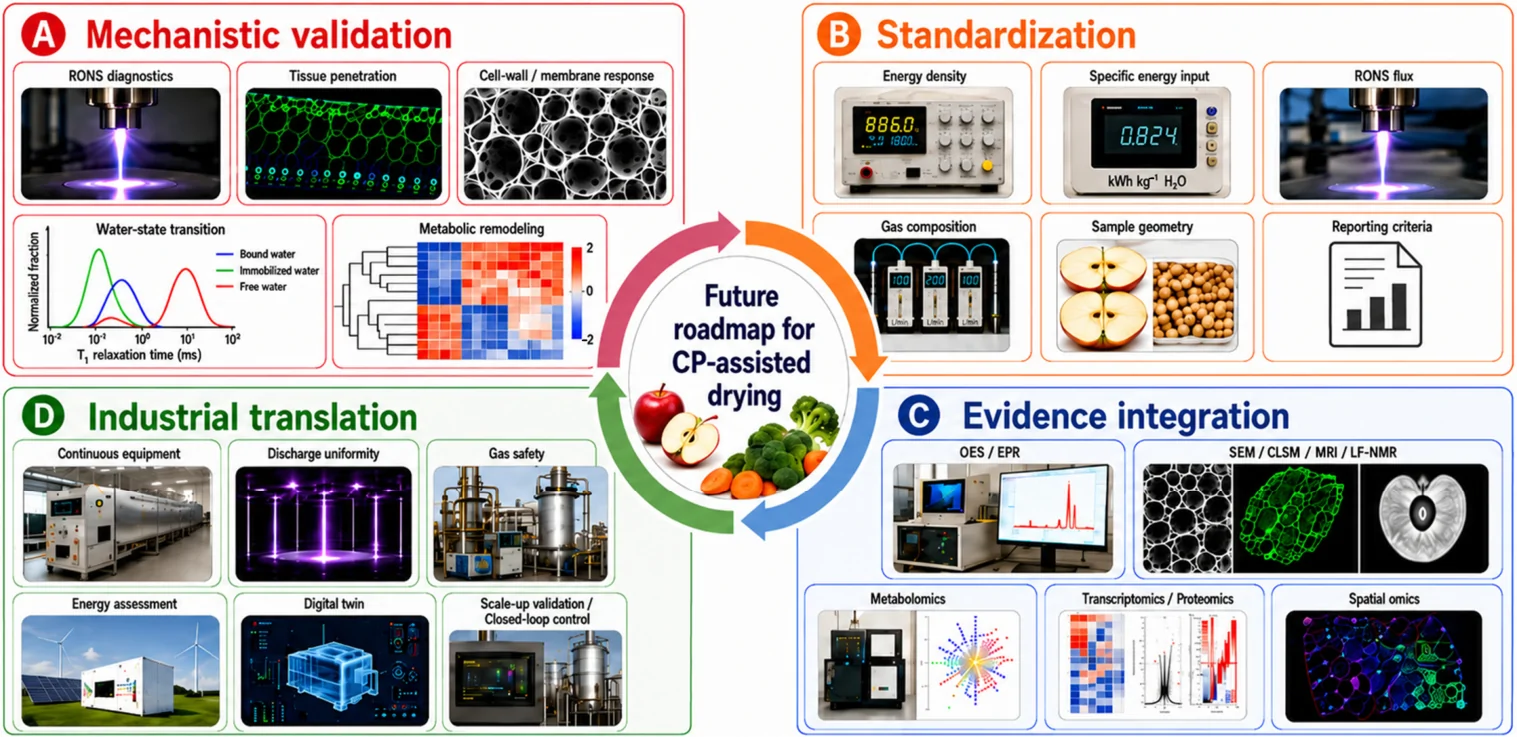
Schematic diagram of a future roadmap for cold plasma (CP) pretreatment-assisted drying of foods, highlighting mechanistic validation, standardization, evidence integration, and industrial translation.

Across current studies, CP pretreatment improves drying only when the gain in interfacial or internal transport exceeds the accompanying oxidative, thermal, or structural burden [43]. Comparable voltage or exposure time can therefore produce faster drying, little change, or quality deterioration as reactor configuration and matrix properties alter the delivered exposure. This balance defines the direction of process development because no single optimum applies across systems. Safety is part of the same processing window because conditions that intensify reactivity may also increase unwanted by-products or quality loss [94]. Figure 5 summarizes the transition from mechanistic understanding to scalable processing.

### 6.1. The Balance Between Transport Enhancement and Quality Deterioration

At low exposure, surface chemistry and tissue structure may change too little to alter the dominant resistance to moisture transfer. Within a productive window, improved wettability, limited etching, cell-wall or membrane loosening, and increased pore connectivity can shorten the drying history while preserving color, texture, nutrients, and flavor [68]. Beyond this window, deeper cracking, leakage, heating, or oxidative attack may outweigh the transport gain. Hybrid and reduced-pressure systems shift these boundaries because mechanical effects, cavitation, gas flow, or pressure-driven moisture removal contribute alongside the plasma treatment [67,147].

The location and width of this window are material-dependent. Surface barriers, cellular architecture, water state, composition, antioxidant capacity, and oxidation-sensitive constituents determine how much of the delivered exposure reaches the dominant transport barrier and how much can be tolerated. A condition that produces limited remodeling in one food may therefore overoxidize another before a meaningful drying benefit appears. The available evidence remains dominated by plant foods and edible fungi [94], and fruit studies illustrate how strongly quality limits depend on the matrix [68]. Extending the scope to all foods therefore requires matrix-specific interpretation, especially for animal-source materials that do not follow plant cell-wall mechanisms.

### 6.2. From Delivered Exposure to Cross-Scale Response

Power and treatment time alone cannot define a matrix-dependent processing window because different exposure configurations deliver different combinations of agents. Direct plasma can couple short-lived RONS with electrons, ions, ultraviolet radiation, electric fields, gas flow, and local heating, whereas afterglow, plasma-processed air, and PAW emphasize transported or persistent chemistry. EHD and other electromagnetic-field treatments can clarify possible physical contributions but remain mechanistic analogs unless plasma chemistry is demonstrated [47,148]. The chemical response is also context-dependent: H_2_O_2_ and NO_x_-derived RNS may participate in signaling, whereas O_3_, •OH, ^1^O_2_, and O_2_•− can increase oxidative burden when antioxidant buffering is exceeded [98,149]. In hybrid systems, mechanical, thermal, and cavitation effects further reshape both exposure and transport [67].

These co-exposures explain why a change in drying rate cannot by itself identify the active mechanism. Faster water removal under reduced pressure may arise without plasma, while a low measured gas temperature does not exclude transient surface or sample heating. Conversely, detecting a species in the discharge does not establish that it reached the food at a structurally or biologically effective dose. Distinguishing these pathways therefore depends on experiments that separate pressure, gas flow, and heating from plasma chemistry [43].

Tracing structural change, moisture redistribution, redox behavior, metabolism, and final quality within the same material would clarify how this sequence unfolds. A productive sequence may begin with interfacial activation and limited tissue remodeling, continue through greater water mobility and a shorter thermal history, and end with preserved redox or metabolic function. The same sequence becomes damaging when etching, membrane failure, or reactive-species accumulation causes leakage, pigment loss, nutrient oxidation, texture collapse, or flavor change. Null responses are also informative because they may indicate that the delivered agents did not reach the dominant transport barrier or that transport gains were offset by damage. Omics studies in broccoli sprouts [102] and blueberries [103] resolve parts of the redox and metabolic response, but they do not yet establish this complete sequence during CP pretreatment-assisted drying.

Different analytical methods resolve different stages of this sequence. Contact-angle measurements and imaging characterize interfacial and structural change, while low-field NMR or MRI follows water redistribution. Enzyme assays and targeted redox analyses probe biochemical responses, and Raman or Vis/NIR spectroscopy provides chemical or quality fingerprints [150,151,152]. Causal interpretation is strengthened when these responses follow the expected temporal and dose-dependent sequence and change under selective perturbation [47]. Multi-omics can reveal coordinated pathways, but mechanistic attribution still requires measured exposure and moisture transport to be linked with drying and quality outcomes in the same experiment [102,103].

### 6.3. Material-Aware Process Design and Industrial Translation

For scale-up, mechanistic knowledge guides the match between reactor and food. A surface-limited material may benefit from controlled interfacial exposure, whereas a matrix dominated by internal diffusion may require deeper remodeling or a complementary pretreatment. Oxidation-sensitive foods may instead favor remote, shorter, or composition-controlled exposure. Broader analysis of nonthermal pretreatments further distinguishes high-sugar matrices, where glass-transition-related stickiness and case hardening remain important, from waxy produce that may respond more directly to cuticular modification [32]. At industrial scale, however, the value of these material advantages depends on whether they persist through changes in exposure uniformity, species transport, thermal load, throughput, emissions, reactive by-products, and storage stability. Sustainability [43] and safety [94] need to be evaluated throughout process development.

Once the relevant matrix and exposure descriptors are defined, data-driven tools can complement mechanistic design. Models trained only on nominal power and treatment time are unlikely to transfer across reactors or foods. By contrast, models that include reactor geometry, species or energy delivery, matrix structure, water state, and quality constraints can identify material-specific operating regions [140]. Transferability then depends on validation with independent batches and equipment scales. Digital twins could extend this approach by linking sensors, mechanistic models, and feedback control, but their industrial value remains prospective until traceability and external validation are demonstrated [142].

Hybrid studies across seeds, roots, leaves, and fruits illustrate how complementary mechanisms can widen the feasible operating region [135,153,154,155,156,157,158]. These combinations are useful when the component processes relieve different limitations without adding a larger quality or energy penalty. This shifts process comparison from the largest reduction in drying time to a system-level balance among water removal, energy use, throughput, product quality, safety, and cost. This system-level perspective links material-specific transport and quality constraints to reactor architecture, exposure chemistry, and complementary processes, which can then be evaluated under continuous or pilot-scale conditions.

## 7. Conclusions

This review synthesizes CP pretreatment-assisted drying of foods through an integrated pathway from delivered chemical and physical agents to structural remodeling, moisture migration, redox regulation, metabolic responses, and final drying and quality outcomes. Across the available evidence, CP is beneficial when surface activation, limited cell-wall or membrane loosening, and greater pore connectivity reduce external or internal mass-transfer resistance. The resulting shorter drying history can limit thermal losses and support color, texture, nutrient, and flavor retention. Excessive etching, leakage, heating, or oxidative attack can instead reverse these gains.

These contrasting outcomes define a material-specific processing window rather than a universal optimum for voltage, power, or treatment time. Its position is jointly governed by reactor configuration and exposure mode, delivered RONS and accompanying physical agents, and the food’s surface barriers, cellular architecture, water state, composition, and antioxidant buffering. This material dependence explains why nominally similar treatments may accelerate drying, produce little effect, or impair quality. It also shifts process design from maximizing a single kinetic response to matching exposure with the dominant transport and quality constraints of each matrix.

Because this interpretation is currently supported mainly by plant-derived foods and edible fungi, extension to animal-source foods requires direct evaluation of matrix-specific transport barriers, water binding, and oxidation-sensitive constituents rather than transfer of plant cell-wall mechanisms. Industrial translation follows the same material-specific principle. Successful scale-up depends on preserving exposure uniformity and transport benefits under continuous or pilot-scale conditions while meeting energy, throughput, product quality, safety, cost, and storage stability requirements. Accordingly, the practical value of CP pretreatment-assisted drying will depend on reproducible operating windows that convert controlled exposure into a favorable balance of drying efficiency and quality for a specified food and process.

## Figures and Tables

**Table 1:** Scope and thematic focus of eight previous reviews and the present review.

Food Materials Covered	Review Approach	Principal Topics	Mechanistic Perspective	Application and Future Directions	Reference
Fruits and vegetables; seven thermal and nonthermal pretreatments, including CP.	Critical narrative review with comparative study tables.	Drying performance, product quality, pretreatment conditions, and reported treatment effects.	Technology-specific effects on mass transfer and quality attributes.	Scale-up, safety, cost, modeling, and process optimization.	Bassey et al. (2021) [20]
Foods; CP pretreatment before drying.	Systematic qualitative review with narrative and tabular synthesis.	Plasma systems, drying efficiency, energy use, product quality, and proposed mechanisms.	Surface and internal structural changes, enzyme responses, and their links to moisture transport.	Equipment, regulation, industrial implementation, and scale-up.	Du et al. (2022) [28]
Foods; CP pretreatment-assisted drying.	Narrative review with a literature overview and study tables.	Drying enhancement, energy saving, product quality, recent advances, and industrial considerations.	Structural changes and the roles of RONS, UV, gas flow, and vacuum.	Equipment, cost, safety, regulation, energy use, and scale-up.	Gavahian et al. (2024) [24]
Fruits and vegetables; CP and other emerging thermal and nonthermal pretreatments.	Critical narrative review.	Drying behavior, bioactive compounds, structural changes, and technology limitations.	Pretreatment-specific structural and bioactive-compound responses.	Commercialization benefits, constraints, and research needs.	Boateng (2024) [29]
Protein-based foods; emerging pretreatments and drying methods.	Narrative review with Web of Science keyword co-occurrence analysis.	Protein structure, functionality, nutritional properties, and drying technologies.	Protein structure-function relationships during pretreatment and drying.	Laboratory-to-industry translation and hybrid processing.	Mahmood et al. (2024) [30]
Fresh-cut fruits and vegetables; multiple pretreatments and hybrid processes.	Narrative synthesis with bibliometric and technology-readiness analyses.	Drying efficiency, product quality, hybrid treatments, and technology maturity.	Effects of individual and hybrid technologies on mass transfer and quality.	Technology-readiness levels, product categories, scale-up, and energy use.	Miletić et al. (2026) [31]
Fruits and vegetables; ultrasound, ozone, PEF, HHP, and CP pretreatments before drying.	Comparative narrative review.	Drying behavior, microstructure, bioactive compounds, and product quality.	Microstructure, moisture migration, and quality responses by pretreatment class.	Cost–benefit considerations, continuous or batch operation, and commodity selection.	Su et al. (2026) [32]
Fruits and vegetables; novel nonthermal pretreatments, including CP.	Systematic review and random-effects meta-analysis of 185 articles, including 21 CP articles.	Drying performance and quality outcomes, pooled effect estimates, and treatment-condition subgroups.	Technology-specific physical and biochemical interpretations of pooled outcomes.	Quantitative evidence for pretreatment selection and qualitative industrial implications.	Zhao et al. (2026) [33]
All edible food materials, with current mechanistic evidence concentrated in plant-derived foods and edible fungi.	Mechanism-centered critical review of CP pretreatment-assisted drying across food materials.	Effects of plasma exposure on structural remodeling, moisture migration, redox regulation, metabolism, drying performance, and product quality.	Links plasma chemistry and physical effects with changes in food structure, moisture migration, redox regulation, and metabolism to explain matrix-dependent outcomes.	Development of material-specific treatment windows and reactor configurations that balance product quality, energy use, safety, and scale-up requirements.	Present review

**Table 2:** Operating characteristics of plasma and related high-voltage systems used in food-drying studies.

Plasma System and Treatment Mode	Food Material and Drying Method	Treatment Environment	Operating Conditions	Plasma Chemistry	Comparative Treatments	Interpretation and Practical Relevance	Reference
Indirect PPA	Apple and potato cubes; HAD 65 °C or freeze-drying	Dry air, 20 L min^−1^; PPA chamber ≈ 22 °C; gas/sample T NR; process pressure NR	Microwave torch ≈ 1.2 kW, 2.45 GHz; 2.5, 5, 7.5 or 10 min; 20 × 5-s ignition/7-s pause	The device specified ≥0.5% NO_2_; under the same stated conditions, a separate experiment reported 2.7% total conversion products; plasma chemistry was not measured during the drying study	Untreated material was compared with the 2.5–10 min PPA treatments; non-plasma processed air was not examined	The PPA was applied remotely; the later 0.5 mbar pressure refers to freeze-drying rather than plasma treatment.	Bußler et al. (2017) [48]
Direct atmospheric CPJ	White grape; HAD 70 °C	Clean dry air, 40 L min^−1^; gas/sample T NR; numerical pressure NR	500 W; 25 kHz; 1 or 5 cm; stage 50 mm s^−1^; each side scanned two or three times	Plasma chemistry was not characterized during the drying experiment	Untreated, skin-removed, chemical-dip, and four CP treatments were compared; gas flow and scanning without plasma were not examined	The waxy intact fruit received CP together with airflow and scanning, so their individual contributions remain unresolved.	Huang et al. (2019) [40]
Direct CPJ and indirect CPAW	Shiitake mushroom; HAD 50–70 °C	Air, 135 L min^−1^; gas/sample T NR; atmospheric treatment; compressor supply 0.2 MPa	650 W; direct CP 1 min (30 s per side), 5 cm; CPAW generated for 10 min	CPAW chemistry was not quantified	Distilled-water immersion control; CPAW; direct CP	Direct and liquid-mediated treatments delivered different exposures; 0.2 MPa denotes gas-supply pressure rather than chamber pressure.	Shishir et al. (2020) [41]
Direct atmospheric CPJ	Wolfberry; HAD 65 °C, 3.0 m s^−1^	Air, 3 L min^−1^; gas/sample T NR; numerical pressure NR	750 W; 20 kHz; 6 cm; 15–60 s	RONS were not characterized during treatment	Untreated; 30 g L^−1^ sodium-carbonate dip	Three trays were dried simultaneously; repeat dryer runs were not described.	Zhou et al. (2020) [49]
Direct atmospheric CPJ	Jujube slices; HAD 50–70 °C	Air, 135 L min^−1^; sample 25.7 °C before and 35.3/48.8/54.2 °C after 15/30/60 s; gas T NR; compressor supply 0.2 MPa	5 kV; 650 W; 40 kHz; 5 cm; 15–60 s per side	RONS information was drawn from an earlier study using the same device at a different voltage and treatment distance	Untreated controls were included at each drying temperature; airflow without plasma was not examined	The measured rise in sample temperature indicates that heating accompanied plasma exposure.	Bao et al. (2021) [50]
Multiple pretreatments, including CP and CPAW	Whole jujube; HAD 70 °C	Air; flow and gas/sample T NR; atmospheric treatment; compressor supply 0.2 MPa	5 kV; 650 W; 40 kHz; 5 cm; 1 min (30 s per side)	RONS were discussed with reference to earlier literature but were not measured in this experiment	Untreated; chemical dip; direct CP; CPAW; ultrasound; thermosonication; blanching	The single-component treatments help distinguish the CP contribution; the stated supply pressure does not represent chamber pressure.	Bao et al. (2022) [51]
Direct low-pressure plasma	Corn kernels; HAD 37.5–52.5 °C	Air, 150 mL min^−1^; sample 24 ± 1 °C before exposure; gas/post-treatment sample T NR; 85 Pa	300–500 W; 40 kHz; 30–50 s; chamber 260 × 260 mm	Plasma chemistry was not measured	Untreated material was used as the control; vacuum exposure without plasma was not examined	Reduced pressure, plasma exposure, and chamber residence may all have contributed to the response.	Li et al. (2019) [52]
Direct DBD	Tucuma pulp; HAD 60 °C	Feed gas and pressure NR; sample surface 28–34 °C after CP; gas T NR	20 kV; 200, 500 or 800 Hz; 10 min; 1.5-cm gap; power NR	Ozone and other reactive species were discussed but not measured	Untreated hot-air drying was used as the reference; discharge-off treatment was not examined	The long exposure produced frequency-dependent oxidation, but the response could not be related to a measured species dose.	Loureiro et al. (2021) [53]
Direct air DBD	Jackfruit slices; HAD 50–70 °C	Air, 2 L min^−1^; surface 25.6 °C untreated and 33.3/38.3/44.7/50.1 °C after 15/30/45/60 s; gas T and pressure NR	Three transformers rated 12 kV, 30 mA, 25 kHz; measured ≈6 kV and ≈1.25 A peak-to-peak, 75% duty; 36.6 W; 15–60 s	OES during treatment detected N_2_ s-positive and first-negative emissions, N, N^+^, and •OH; concentrations were not quantified	Untreated controls were included at each drying temperature; airflow without plasma was not examined	OES identified emitting species without quantifying their concentration or delivery to the tissue; sample heating increased at longer exposures.	Seelarat et al. (2024) [54]
Direct low-pressure CP	Mango; HAD 60 °C	Process gas NR; no detectable surface/core temperature change, absolute T NR; 1.4 mbar	90 kV; 5 or 10 min; power, frequency and flow NR	Reactive species were proposed as part of the mechanism but were not measured	Untreated and 1% sodium metabisulfite treatments were compared; vacuum exposure without plasma was not examined	The response may reflect both reduced pressure and plasma exposure.	Yanclo et al. (2024) [55]
EHD analogue	Shiitake mushroom; EHD drying	Gas, temperature and pressure NR	AC 0, 18, 22, 26, 30 or 34 kV; frequency/power NR	The study focused on ionic wind and did not characterize reactive species	0 kV control	The findings describe EHD-enhanced convection rather than a RONS-mediated CP mechanism.	Xiao et al. (2022) [56]
EHD analogue	Apple and strawberry slices; EHD drying	20 ± 2 °C, 60% RH; ambient, numerical pressure NR; process gas NR	22 kV DC; 90.8–160.5 µA; 16.8–18.6 W	The study focused on ionic-wind and electric-field effects and did not characterize reactive species	Compared with hot-air drying; an ambient 0-kV treatment was not included	This low-temperature EHD study illustrates physical drying enhancement, although the contribution of ambient convection was not isolated.	Paul et al. (2022) [57]

Note: NR, not reported; PPA, plasma-processed air; CPAW, cold-plasma-activated water; CPJ, cold plasma jet; DBD, dielectric barrier discharge; EHD, electrohydrodynamic; HAD, hot-air drying; OES, optical emission spectroscopy.

**Table 3:** Effects of CP pretreatment on drying performance, product quality, and related mechanisms in food materials.

Food Material and Treatment Mode	Treatment and Drying Conditions	Comparison Groups	Experimental Design and Analysis	Drying Endpoint	Drying Performance	Quality and Mechanistic Findings	Reference
White grape; direct CPJ	Dry air; 500 W; 25 kHz; 40 L min^−1^; 1 or 5 cm; two/three passes per side; HAD 70 °C	Untreated; skin-removed; 0.5% NaOH + 2% ethyl oleate; four CP arms	At least three drying experiments; aw measured in three raisins; color and texture assessed in ≥3 samples at three points per sample. One-way ANOVA (CoStat, *p* < 0.05); post-hoc test not stated	Equilibrium (no further mass change); numerical moisture content not stated; aw 0.52–0.55 across treatments	Plasma response strengthened with shorter nozzle distance and repeated passes. Drying-enhancing treatments reached final moisture in <25 h, 15–20% sooner than untreated; 1 cm/three passes was comparable to the skin-removed and chemical controls.	Surface etching, cracks, and quality changes were reported, but the study did not measure RONS.	Huang et al. (2019) [40]
Grape; direct gliding arc	Air; 27 kV; 300 W; 50 Hz; 10 L min^−1^; 10–60 s; HAD 60 °C, 1.5 m s^−1^	Untreated 0-s control	Drying and quality measurements were performed in triplicate. One-way ANOVA with Fisher LSD (Minitab 19, *p* < 0.05) and Pearson correlation	Target moisture content 12% wet basis; aw not stated	Across 10–60 s, drying-time reductions ranged from 4.71% to 26.27%; the benefit increased through 50 s but partially reversed at 60 s. At 50 s, energy consumption during drying fell from 45.72 to 33.70 kWh (−26.30%).	OES qualitatively detected NO•, excited N_2_, and •OH without concentration measurements; quality and rehydration responses depended on exposure time.	Ashtiani et al. (2020) [74]
Wolfberry; direct CPJ	Air; 750 W; 20 kHz; 3 L min^−1^; 6 cm; 15–60 s; HAD 65 °C	Untreated; 30 g L^−1^ sodium-carbonate dip	Three 150-g groups per condition were dried simultaneously on three trays; color *n* = 15; other assays in triplicate. ANOVA with Duncan’s test or Kruskal–Wallis, as applicable (SPSS 25)	0.15 g H_2_O g^−1^ dry mass; aw not stated	Untreated: 14 h; CP 15 s: 9 h (−35.7%); CP 30, 45, and 60 s: 7 h each (−50.0%).	The study reported micro- and ultrastructural changes, rehydration, color, and polyphenols but did not measure RONS.	Zhou et al. (2020) [49]
Jujube slices; direct CPJ	Air; 5 kV; 650 W; 40 kHz; 135 L min^−1^; 5 cm; 15–60 s per side; HAD 50–70 °C	Untreated control at each drying temperature	Drying was measured in triplicate across ≥3 independent experiments. One-way ANOVA with Duncan’s test (SPSS 22, *p* < 0.05)	Constant mass; numerical moisture content and aw not stated	D_eff_ (×10^−10^ m^2^ s^−1^; control/15/30/60 s) was 2.21/1.91/1.89/1.52 at 50 °C, 3.58/4.34/3.55/3.32 at 60 °C, and 3.79/6.76/4.26/3.25 at 70 °C, showing a temperature-dependent, non-monotonic response.	The study reported cavities and bioactive quality changes; the sample surface reached 54.2 °C at 60 s, and RONS data were taken from an earlier experiment.	Bao et al. (2021) [50]
Chili pepper; direct gliding arc	Air; 750 W; 20 kHz; 3 L min^−1^; 6 cm; 15–60 s per side; impingement drying 70 °C, 6.0 m s^−1^	Untreated hot-air-dried control	Experiments were performed in triplicate; color ≥ 15 measurements per group; pigment assays in triplicate. ANOVA with Duncan’s test (SPSS 21, *p* < 0.05)	Target moisture content 10% wet basis; aw not stated	All CP groups dried faster than the untreated control, but the response was non-monotonic: 30 s gave the minimum drying time (5 h), whereas 45–60 s did not improve it further.	Microholes were observed; red pigment peaked at 30 s, whereas antioxidant activity increased with exposure. RONS were not measured.	Zhang et al. (2019) [124]
Corn kernels; low-pressure plasma	Air; 85 Pa; 300–500 W; 40 kHz; 30–50 s; HAD 37.5–52.5 °C	Untreated material was used as the control; vacuum exposure without plasma was not examined	Experiments were performed at least in duplicate, with triplicate drying measurements and three AFM images per sample. One-way ANOVA with Duncan’s test (SPSS, *p* < 0.05)	Final moisture content 13.5 ± 0.2%; basis not restated; aw not stated	At 500 W/30 s, control-to-CP drying times were 4.75 to 3.75 h at 37.5 °C (−21.05%; abstract: −21.52%), 3.00 to 2.50 h at 45 °C (−16.67%), and 2.00 to 1.75 h at 52.5 °C (−12.50%). At 45 °C, 30–50 s all reached 2.5 h, with no further time reduction.	AFM roughness increased. RONS were not measured, and reduced pressure may have contributed to the response.	Li et al. (2019) [52]
Shiitake mushroom; direct CPJ and CPAW	Air; 650 W; 135 L min^−1^; direct CP 60 s; 5 cm; CPAW 10 min; HAD 50–70 °C	Distilled-water immersion; CPAW; direct CP	Drying and analytical measurements were performed in triplicate (*n* = 3). One-way ANOVA with Duncan’s test (SPSS 17, *p* < 0.05)	Target moisture content 10% wet basis; aw not stated	At 50, 60, and 70 °C, direct CP reached the endpoint before CPAW and the distilled-water control; treatment-wise numerical reductions were not reported in the text (Figure 3 only). Starting moisture differed (>90% for control/CPAW versus approximately 84% for direct CP).	The study reported surface and cell-wall changes, sugars, vitamins, phenolic acids, and antioxidant outcomes. It is a follow-up report from the experimental series described in [41].	Karim et al. (2021) [125]
Tucuma pulp; direct DBD	20 kV; 200, 500 or 800 Hz; 1.5-cm gap; 10 min; HAD 60 °C	Untreated hot-air-dried control	Measurements were performed in triplicate. One-way ANOVA with Duncan’s test (R 3.6.0, 95% confidence)	Final moisture: untreated 11 ± 2 and CP treatments 5 ± 1 to 8.3 ± 0.5 g water per 100 g fruit; aw not stated	Untreated: 270 min; 200 Hz: 105 min (−61.1%); 500 Hz: 210 min (−22.2%); 800 Hz: 120 min (−55.6%), demonstrating a non-monotonic frequency response.	Cavities and quality responses varied with frequency; the sample reached 28–34 °C. RONS were not measured.	Loureiro et al. (2021) [53]
Tomato peel; direct atmospheric CAP	Air; point-to-plate; 50 µs pulses; 100 Hz; ≤20 kV; 8 kV cm^−1^; 6000 discharges; 1.7 kJ kg^−1^; IR drying	Protocol D: untreated peel + IR drying; protocol C: CAP-treated peel + IR drying	At least five replicates were analyzed. ANOVA with LSD (SigmaPlot 14, *p* < 0.05) and pairwise Tukey tests (α = 0.05)	≈0.06 g per 100 g dry matter, as reported; aw not stated	Control versus CAP: 143 versus 119 min (−18.2%); specific energy consumption of the overall process was 4.5 ± 0.33 versus 3.7 ± 0.21 kW kg^−1^ (approximately −17%).	The sample-control temperature difference was <2 °C, and electroporation with dendritic-channel formation was proposed; RONS were not measured.	Sosnin et al. (2023) [126]
Apple slices; direct atmospheric CP	25 kV; 50 Hz; total 5–20 min (2.5–10 min per side); 3 cm; RWD water 90 °C or HAD 70 °C	Untreated RWD and untreated HAD controls	Experiments were repeated three times; moisture *n* = 3. One-way ANOVA with Tukey’s test (JMP Pro 14.0, *p* < 0.05)	Target moisture content 10% wet basis; aw not stated	At 10 min, RWD decreased from 70 to 55 min (−21.4%) and HAD from 420 to 330 min (−21.4%). The 5- and 20-min groups were intermediate; 20 min remained faster than control but slower than 5–10 min (Figure 3).	The study reported pores, PPO inactivation, and bioactive compounds. RONS and CP sample temperature were not measured; RWD product temperature was 69 ± 1 °C.	Subrahmanyam et al. (2024) [62]
Mango cultivars; direct low-pressure CP	O_2_; 90 kV; 1.4 mbar; 5 or 10 min; HAD 60 °C	Untreated drying and 1% sodium metabisulfite treatments were used as references; vacuum-only treatment was not examined	Experiments were repeated six times (most outcomes *n* = 6); bioactive assays were performed in triplicate and microbial measurements used *n* = 9. GLM/factorial ANOVA with Fisher LSD (SAS 9.4)	Target moisture content 10.0% wet basis; aw not stated	Drying times (control/SMB/CP5/CP10) were 12/12/10/10 h for Tropica and 10/10/9/9 h for Keitt, demonstrating cultivar-dependent responses.	Structure and bioactive responses differed by cultivar. RONS were not measured, and absolute gas and sample temperatures were not stated.	Yanclo et al. (2024) [42]
Green pea; direct DBD	20 kV; 250–1000 Hz; duty 30–90%; 30–120 s; thin-layer HAD	Untreated hot-air-dried control	Treatments were performed in triplicate; color *n* = 6. One-way ANOVA with Duncan’s test (*p* < 0.05) and Spearman correlation	Final moisture content 12.00% wet basis; aw not stated	Control: 330 min. At 30/60/90/120 s: 300/280/270/270 min; at 250/500/750/1000 Hz: 270/270/270/300 min; at 30/50/70/90% duty: 300/300/270/270 min. D_eff_ increases ranged from 7.72% to 66.31% across settings.	Micropores and increased roughness were reported, together with 24.06% higher total phenolics and 29.64% higher antioxidant activity; RONS were discussed but not measured.	Bai et al. (2025) [76]

## Data Availability

No new data were created or analyzed in this study. Data sharing is not applicable to this article.

## Supplementary Materials

The following supporting information can be downloaded at: https://www.mdpi.com/article/10.3390/foods15162887/s1, Table S1: Web of Science Core Collection search strategy, screening summary, and eligibility criteria; Table S2: Study-level characteristics of the primary evidence included in this review [159,160,161,162,163,164,165,166,167,168,169,170,171,172,173,174,175,176,177,178,179,180,181,182,183,184,185,186,187,188,189,190,191,192,193,194,195,196,197,198,199,200,201,202,203,204,205,206,207,208,209,210,211,212,213,214,215].
