# Supplementary material for: Structural Remodeling, Redox Regulation, and Metabolic Responses in Cold Plasma Pretreatment-Assisted Drying of Foods: A Critical Review
Source: Foods. 2026 Aug 18;15(16):2887. doi: 10.3390/foods15162887 (PMC13512583; doi:10.3390/foods15162887)
Supplement: Supplementary file 1 [file foods-15-02887-s001.zip › foods-4446093-supplementary.pdf]

Supplementary Materials

Structural Remodeling, Redox Regulation, and Metabolic Responses in Cold-Plasma Pretreatment-Assisted Drying of Foods: A Critical Review

Kai Zhang [1], Qingqing Yuan [1], Tianrui Liu [1], Lilang Li [2], Zhou He [3], Shi Jianyong [3], Roujia Zhang [1], Siyao Liu [4], Yu Wang [2,*], Chenguang Zhou [1,*]

Table S1. Web of Science Core Collection search strategy, screening summary, and eligibility criteria.

Item	Specification
**Database and search date**	Web of Science Core Collection; searched on 10 August 2026, with coverage from database inception to the search date.
**Search field**	Topic search (TS), covering title, abstract, author keywords, and Keywords Plus.
**Search query**	`TS=(("cold plasma" OR "cold atmospheric plasma" OR "nonthermal plasma" OR "non-thermal plasma" OR "low-temperature plasma" OR "low temperature plasma" OR "atmospheric-pressure plasma" OR "atmospheric pressure plasma" OR "dielectric barrier discharge" OR "plasma jet" OR "gliding arc plasma" OR "plasma-processed air" OR "plasma processed air" OR "plasma-activated water" OR "plasma activated water") AND (drying OR dehydration OR dewatering))`
**Limits**	Document type: Article or Review Article; language: English.
**Screening summary**	The search retrieved 1,711 records; 1,705 remained after document-type filtering and 1,695 after language filtering. Title-and-abstract screening retained 255 records for detailed eligibility assessment. The final evidence base comprised 85 primary studies, while 8 reviews were retained for field positioning and comparison.
**Primary-study inclusion criteria**	Studies were included when they investigated edible materials and applied a direct, indirect, or hybrid cold-plasma-related treatment before or during drying. At least one drying, moisture-transfer, energy, or dried-product-quality outcome was required.
**Exclusion criteria**	Studies were excluded from the primary evidence base if they examined non-edible materials, applied the plasma-related treatment only after completion of drying, or reported none of the prespecified drying, moisture-transfer, energy, or dried-product-quality outcomes. Studies focused solely on sterilization, germination, extraction, or other non-drying endpoints were likewise excluded from the primary evidence set, although selected non-drying studies were retained as clearly identified mechanistic references where relevant.

Table S2. Study-level characteristics of the primary evidence included in this review.

Food material	Plasma or treatment system	Drying method	Evidence reported	Primary outcome(s)	Evidence classification	Reference
Apple slices	Cold plasma	Vacuum-microwave drying	Moisture transfer/water state; Microstructure; Product quality; Biochemical/structural response	Longer CP exposure increased surface etching and moisture migration; CP also reduced PPO/POD, increased PAL, and improved retention of phenolics during storage of vacuum-microwave-dried apple chips.	Direct	[22]
White grape	Atmospheric-pressure air plasma jet	Hot-air drying	Drying kinetics; Moisture transfer/water state; Energy/environmental performance; Product quality	The atmospheric plasma jet etched the waxy grape cuticle, shortened drying by more than 20%, increased hydrophilicity, and improved phenolic content and antioxidant capacity without major changes in appearance or texture.	Direct	[40]
Shiitake mushroom	Direct cold plasma and cold plasma-activated water	Hot-air drying	Drying kinetics; Moisture transfer/water state; Microstructure; Energy/environmental performance; Product quality; Modeling/process prediction	Direct CP and CPAW both modified shiitake microstructure, accelerated drying, reduced energy requirements, and improved retention of phenolics, flavonoids, and antioxidant activity.	Direct/Indirect	[41]
Tropica and Keitt mangoes	Low-pressure cold plasma	Hot-air drying	Drying kinetics; Energy/environmental performance; Product quality; Microbial safety; Modeling/process prediction	Low-pressure CP improved mango drying rate, phenolics, and antioxidant activity, while cultivar significantly influenced drying time and other responses.	Direct	[42]
Apple and potato tissues	Plasma-processed air	Warm-air and freeze-drying	Product quality; Biochemical/structural response; Microbial safety	Plasma-processed air reduced PPO and POD activities in apple and potato. Product responses were matrix-dependent, including prevention of potato blackening but atypical browning in apple.	Indirect	[48]
Wolfberry	Cold plasma	Hot-air drying	Drying kinetics; Microstructure; Product quality	CP shortened wolfberry drying and improved rehydration and color, while phytochemicals first increased and then decreased as exposure time increased.	Direct	[49]
Jujube slices	Cold plasma	Hot-air drying	Drying kinetics; Moisture transfer/water state; Microstructure; Product quality; Modeling/process prediction	CP etched the jujube surface, increased moisture transfer, improved phenolics and antioxidant capacity, and reduced 5-hydroxymethylfurfural formation.	Direct	[50]
Whole jujube	Cold plasma and cold plasma-activated water; non-plasma pretreatments as comparators	Hot-air drying	Drying kinetics; Microstructure; Product quality	Among the green pretreatments compared, direct CP altered the jujube surface, shortened drying, enhanced phenolics and antioxidant capacity, and reduced 5-hydroxymethylfurfural formation.	Direct/Indirect	[51]
Corn kernels	Low-temperature plasma	Hot-air drying	Drying kinetics; Moisture transfer/water state; Microstructure; Energy/environmental performance	Plasma pretreatment shortened hot-air drying and increased drying rate, moisture diffusivity, and surface porosity, with the largest kinetic benefit reported at the lower drying temperature.	Direct	[52]
Tucuma pulp	Cold plasma at different excitation frequencies	Hot-air drying	Drying kinetics; Product quality	Excitation frequency produced a non-monotonic response: 200 and 800 Hz shortened drying with little color change, whereas carotenoid degradation was greatest at 500 Hz.	Direct	[53]
Jackfruit	DBD plasma	Hot-air drying	Drying kinetics; Moisture transfer/water state; Microstructure; Product quality; Modeling/process prediction	DBD pretreatment promoted moisture transfer and bioactive retention in dried jackfruit, but color and carotenoid levels declined.	Direct	[54]
Heidi mango	Low-pressure cold plasma	Hot-air drying	Drying kinetics; Microstructure; Product quality; Microbial safety; Modeling/process prediction	Low-pressure CP shortened mango drying and improved selected phenolic and color outcomes, but the longer exposure yielded the lowest antioxidant activity; microbial load decreased by at least 2 log.	Direct	[55]
Apple slices	Cold plasma; ultrasound and microwave pretreatment comparators	Hot-air drying	Drying kinetics; Moisture transfer/water state; Microstructure; Energy/environmental performance; Product quality	CP surface etching shortened apple drying and reduced energy use while improving water migration, moisture uniformity, color, morphology, and rehydration relative to the untreated control.	Direct	[58]
Apple slices	Multipin atmospheric-pressure cold plasma	Refractance-window drying	Drying kinetics; Microstructure; Product quality; Biochemical/structural response	Multipin CP accelerated refractance-window drying, improved rehydration and texture, reduced PPO activity, and increased retention of antioxidants and bioactive compounds.	Direct	[62]

Food material	Plasma or treatment system	Drying method	Evidence reported	Primary outcome(s)	Evidence classification	Reference
Fresh wet noodles	Cold plasma dehydration treatment	Plasma-assisted partial dehydration	Product quality	Cold plasma rapidly removed part of the water from fresh wet noodles and increased whiteness, hardness, and structural stability while redistributing protein secondary structures.	Direct	[63]
Grape	Gliding-arc plasma	Hot-air drying	Drying kinetics; Moisture transfer/water state; Energy/environmental performance; Product quality	Gliding-arc pretreatment increased moisture diffusivity and reduced drying time and energy use; quality responses, including phenolics, antioxidant activity, and vitamin C retention, varied with exposure time.	Direct	[74]
Saffron	Gliding-arc discharge plasma	Hot-air drying	Drying kinetics; Energy/environmental performance; Product quality	Longer gliding-arc exposure shortened drying and reduced energy consumption; intermediate exposure better retained crocin, picrocrocin, phenolics, and antioxidant activity.	Direct	[75]
Chili pepper	Cold plasma	Hot-air drying	Drying kinetics; Moisture transfer/water state; Microstructure; Product quality	CP enhanced chili drying and antioxidant activity, with an intermediate exposure giving the best drying and pigment retention and longer exposure negatively affecting red pigment.	Direct	[124]
Shiitake mushroom	Cold plasma	Hot-air drying	Microstructure; Product quality	CP-induced cellular disruption accelerated shiitake drying and improved powder functionality and retention of sugars, vitamins, phenolic acids, and antioxidant activity.	Direct	[125]
Tomato peel	Cold atmospheric plasma	Infrared drying	Energy/environmental performance	CAP pretreatment improved tomato peelability and shortened infrared peel drying while reducing specific energy consumption.	Direct	[126]
Button mushroom (Agaricus bisporus)	Cold plasma coupled with osmotic dehydration	Osmotic dehydration followed by convective drying	Drying kinetics; Moisture transfer/water state; Microstructure; Energy/environmental performance; Product quality	CP followed by osmotic dehydration increased moisture and calcium transfer and reduced convective drying time and energy use while improving several nutritional and texture attributes.	Hybrid	[127]
Caraway seeds	Cold plasma followed by ultrasound during drying	Ultrasound-assisted hot-air drying	Energy/environmental performance; Product quality; Modeling/process prediction	Combined CP and ultrasound reduced drying time and specific energy use and improved phenolics and antioxidant capacity; response-surface optimization identified a balanced operating condition.	Hybrid	[135]
Chinese yam	Corona-discharge and DBD plasma; ultrasound comparator	Hot-air drying	Drying kinetics; Moisture transfer/water state; Energy/environmental performance; Product quality; Modeling/process prediction	The study compared two plasma modes with ultrasound. Ultrasound produced the strongest drying and quality response, while the plasma treatments provided separate CP-specific evidence.	Direct	[153]
Beetroot	Cold plasma	Intermittent microwave-hot-air drying	Drying kinetics; Moisture transfer/water state; Microstructure; Energy/environmental performance; Product quality	Longer CP exposure increased drying rate and diffusivity and reduced specific energy use, shrinkage, and bulk density while increasing rehydration in intermittent microwave-hot-air drying.	Direct	[154]
Centella asiatica leaves	DBD-generated cold plasma-activated water (CPAW)	Microwave drying	Drying kinetics; Product quality; Modeling/process prediction	CPAW altered microwave-drying kinetics and quality; the Page and Logarithmic models provided the best fits, and the reported optimum combined 540 W with about 7 min of drying.	Indirect	[155]
Cumin seeds	Cold plasma followed by ultrasound during drying	Ultrasound-assisted air drying	Drying kinetics; Moisture transfer/water state; Microstructure; Energy/environmental performance; Product quality	CP pretreatment and ultrasound-assisted drying jointly reduced drying time and energy use; the longer CP exposure produced a denser surface and less favorable color and rupture-force responses.	Hybrid	[156]
Goldenberry	Cold plasma followed by ultrasound-assisted drying	Ultrasound-assisted convective drying	Drying kinetics; Moisture transfer/water state; Energy/environmental performance; Product quality	CP enabled lower-temperature processing, while ultrasound reduced convective drying time; the combined sequence improved vitamin C, phenolics, antioxidant activity, and shelf stability.	Hybrid	[157]
Kiwifruit	Plasma-activated water and plasma jet, alone or combined with ultrasound	Vacuum freeze-drying	Microstructure; Product quality	PAW/plasma-jet pretreatments, particularly when combined with ultrasound, increased porosity and improved moisture, rehydration, texture, and color outcomes, although antioxidant capacity decreased in all pretreated groups.	Direct/Indirect/Hybrid	[158]
Wolfberry (goji berry)	PEF-driven dielectric barrier discharge (DBD) plasma; APPJ and PEF comparators	Hot-air drying	Drying kinetics; Microstructure; Energy/environmental performance; Product quality; Biochemical/structural response	PEF-driven DBD reduced drying time and energy consumption, while surface etching, tissue disruption, and altered water binding were associated with improved moisture transfer and quality retention.	Direct	[159]

Food material	Plasma or treatment system	Drying method	Evidence reported	Primary outcome(s)	Evidence classification	Reference
Centella asiatica leaves	DBD-generated cold plasma-activated water (CPAW)	Recirculatory hot-air and vacuum drying	Drying kinetics; Microstructure; Product quality; Modeling/process prediction	CPAW pretreatment improved drying behavior and quality relative to blanching, with the best preservation of microstructure and bioactives reported under vacuum drying at the lower tested temperature.	Indirect	[160]
Saimaiti apricot	Atmospheric-pressure plasma jet (APPJ)	Heat-pump drying	Drying kinetics; Moisture transfer/water state; Microstructure; Energy/environmental performance; Product quality; Biochemical/structural response	APPJ shortened heat-pump drying and improved porosity, moisture transport, color, bioactive retention, and aroma preservation compared with the tested alternatives.	Direct	[161]
Chinese yam	Plasma-activated water combined with ultrasound	Electrohydrodynamic drying	Product quality; Biochemical/structural response	Ultrasound-assisted PAW produced faster EHD drying and improved rehydration, color, sugars, phenolics, and volatile retention relative to the water-based control.	Hybrid	[162]
Ginger	Cold plasma jet and DBD plasma	Hot-air drying	Drying kinetics; Microstructure; Product quality; Microbial safety; Modeling/process prediction	Short CPJ or DBD treatments increased moisture diffusivity and improved bioactive and antibacterial responses; longer DBD exposure reduced surface pore area and was associated with lower diffusivity.	Direct	[163]
Agar gel (model food)	Multipin DBD plasma	DBD/EHD plasma drying	Drying kinetics; Modeling/process prediction	Higher electric-field strength in the multipin DBD configuration increased the drying rate of agar gel, supporting an electrohydrodynamic contribution to plasma drying.	Direct	[164]
Blueberry	Gliding-arc discharge low-temperature plasma	Convective hot-air drying	Drying kinetics; Moisture transfer/water state; Microstructure; Product quality	Gliding-arc pretreatment reduced drying time and increased moisture diffusivity and rehydration. Phenolics improved, whereas color darkened and texture changed with treatment intensity.	Direct	[165]
Lettuce leaves	Plasma-activated water	Oven drying	Modeling/process prediction	PAW-treated and control lettuce followed similar modelled moisture-loss patterns; the study did not establish an unequivocal PAW effect on drying dynamics.	Indirect	[166]
Beetroot	DBD cold atmospheric plasma	Intermittent microwave-hot-air drying	Drying kinetics; Microstructure; Product quality; Biochemical/structural response	DBD pretreatment increased porosity and shortened intermittent microwave-hot-air drying; the conditions maximizing drying efficiency differed from those maximizing phytochemical retention.	Direct	[167]
Plum	Gliding-arc plasma	Hot-air drying	Drying kinetics; Moisture transfer/water state; Microstructure; Energy/environmental performance	Gliding-arc treatment increased permeability and redistributed water, substantially shortening drying in laboratory tests; factory-scale testing also indicated lower emissions and a short payback period.	Direct	[168]
Carrot	Cold plasma jet	Vacuum and convective tray drying	Drying kinetics; Moisture transfer/water state; Microstructure; Energy/environmental performance; Product quality; Biochemical/structural response	Cold plasma shortened vacuum and tray drying, increased diffusivity, and reduced energy use and estimated CO2 emissions while altering carrot structure and improving several quality indices.	Direct	[169]
Barberry	DBD and gliding-arc plasma	Hot-air and microwave drying	Drying kinetics; Moisture transfer/water state; Microstructure; Product quality; Modeling/process prediction	DBD and gliding-arc pretreatments shortened hot-air and microwave drying and altered quality and hyperspectral responses; intensive treatment reduced anthocyanins despite gains in diffusivity and antioxidant extractability.	Direct	[170]
Debittered sweet orange peel	Plasma-activated water	Hot-air tray drying	Drying kinetics; Product quality; Biochemical/structural response; Modeling/process prediction	PAW-treated and control samples both dried faster as temperature increased. The PAW-treated powder showed greater porosity and crystallinity and altered functional properties, with the reported optimum at 60 degrees C and 0.9 m/s.	Indirect	[171]
Heidi mango	Low-pressure cold plasma and plasma-jet treatment	Hot-air drying	Biochemical/structural response; Microbial safety	Cold-plasma treatment altered mango-associated bacterial communities, and the combination of plasma-jet pretreatment and drying eliminated detectable Cyanobacteria in the dried slices.	Direct	[172]
Sweet orange peel	Plasma-activated water	Hot-air drying	Product quality; Biochemical/structural response	PAW soaking increased phenolics, flavonoids, antioxidant activity, and relative crystallinity while reducing antinutritional compounds in hot-air-dried sweet orange peel powder.	Indirect	[173]
Pepper, lentil, and pea	Low-temperature plasma	Hot-air drying	Drying kinetics; Moisture transfer/water state; Energy/environmental performance; Product quality	Plasma pretreatment shortened drying and increased moisture diffusivity in pepper, lentil, and pea, although extending the plasma duration did not consistently provide further improvement.	Direct	[174]

Food material	Plasma or treatment system	Drying method	Evidence reported	Primary outcome(s)	Evidence classification	Reference
Clitoria ternatea flowers	DBD cold plasma	Hot-air drying	Drying kinetics; Energy/environmental performance; Product quality; Modeling/process prediction	DBD pretreatment increased drying rate, bioactive retention, and antioxidant activity and lowered the reported carbon footprint across the tested voltage range.	Direct	[175]
Carrot	Cold plasma jet	Vacuum and convective tray drying	Drying kinetics; Biochemical/structural response; Modeling/process prediction	Hybrid machine-learning and optimizer-informed empirical models predicted moisture dynamics across plasma exposure and vacuum/tray drying conditions; the empirical digital twin provided the best interpretability and prediction.	Direct	[176]
Goji berry	DBD plasma; freezing-thawing, ultrasound, and PEF comparators	Hot-air drying	Drying kinetics; Moisture transfer/water state; Microstructure; Energy/environmental performance; Product quality; Biochemical/structural response; Modeling/process prediction	Among four physical pretreatments, DBD produced the greatest reduction in drying time and energy use and improved browning control through surface disruption and changes in water state.	Direct	[177]
Lotus pollen	DBD plasma	DBD plasma drying	Drying kinetics; Moisture transfer/water state; Microstructure; Energy/environmental performance; Product quality	Increasing DBD voltage greatly shortened lotus-pollen drying and improved color while changing phenolic-acid composition; total and net energy measures differed by several orders of magnitude.	Direct	[178]
Canola seeds	DBD cold plasma	Fluidized-bed drying	Drying kinetics; Moisture transfer/water state; Product quality; Modeling/process prediction	Moderate plasma exposure shortened fluidized-bed drying and increased oil yield at lower temperatures, whereas prolonged exposure at the highest temperature slightly prolonged drying and impaired color/oil responses.	Direct	[179]
Carrot	DBD plasma	Hot-air drying	Drying kinetics; Moisture transfer/water state; Product quality	DBD pretreatment increased moisture diffusivity and shortened carrot drying, while color, carotenoids, phenolics, polysaccharides, and volatile profiles responded differently across exposure times.	Direct	[180]
Fresh-cut Chinese yam	Cold plasma	Hot-air drying	Drying kinetics; Moisture transfer/water state; Energy/environmental performance; Product quality; Biochemical/structural response; Modeling/process prediction	CP reduced POD activity through protein-structural modification and accelerated dehydration, with the drying and anti-browning optima occurring at different treatment durations.	Direct	[181]
Tomato slices	Plasma-treated water combined with osmotic dehydration	Infrared-assisted pulsed-vacuum drying	Drying kinetics; Moisture transfer/water state; Energy/environmental performance; Product quality	Plasma-treated water combined with osmotic dehydration increased moisture diffusivity and shortened drying while improving several bioactive and sensory attributes, but rehydration and hardness decreased relative to the control.	Indirect/Hybrid	[182]
Keitt mango	Cold plasma	Hot-air drying	Product quality; Microbial safety	After storage and drying, CP-treated mango retained more flavanols than the dried untreated control and showed at least a 2-log microbial reduction, although yellowness increased during storage and drying.	Direct	[183]
Chinese yam	Cold plasma	Far-infrared drying	Drying kinetics; Moisture transfer/water state; Energy/environmental performance; Product quality	CP pretreatment shortened far-infrared drying, reduced energy use, promoted outward moisture migration, and improved color and bioactive retention under the reported balanced condition.	Direct	[184]
Jujube slices	Cold plasma; ultrasound, ultrahigh pressure, and PEF comparators	Microwave-coupled pulsed-vacuum drying	Drying kinetics; Microstructure; Product quality; Biochemical/structural response	CP, ultrasound, and PEF each shortened microwave-pulsed-vacuum drying; CP-treated jujube showed the highest flavonoid content among the tested pretreatments.	Direct	[185]
Wheat flour and whey protein isolate	Atmospheric cold plasma	Atmospheric-plasma dehydration	Drying kinetics; Product quality; Microbial safety; Modeling/process prediction	Atmospheric plasma provided rapid dehydration and coliform inactivation in wheat flour and whey protein isolate; functional-property responses were material- and endpoint-dependent.	Direct	[186]
Corn kernels	Cold plasma	Hot-air drying	Drying kinetics; Microstructure; Energy/environmental performance; Modeling/process prediction	Optimization identified high plasma power, longer treatment, and the highest drying temperature as the fastest corn-drying condition; plasma-created surface microholes supported the kinetic response.	Direct	[187]
Pomegranate peel	Cold plasma; microwave pretreatment comparator	Hot-air drying	Drying kinetics; Microstructure; Energy/environmental performance; Product quality	CP pretreatment altered peel microstructure and accelerated hot-air drying while preserving chemical, physical, nutritional, and color attributes better than the tested microwave pretreatment.	Direct	[188]

Food material	Plasma or treatment system	Drying method	Evidence reported	Primary outcome(s)	Evidence classification	Reference
Yellow Delicious apple	Cold plasma with laminar airflow (numerical simulation)	Plasma-air drying (numerical simulation)	Drying kinetics; Energy/environmental performance; Modeling/process prediction	The simulation predicted little drying effect from plasma without airflow, but a large reduction in drying time and energy consumption when plasma was combined with heated laminar airflow.	Direct	[189]
Haskap berry	Cold plasma	Vacuum freeze-drying	Drying kinetics; Microstructure; Energy/environmental performance; Product quality; Biochemical/structural response; Modeling/process prediction	CP increased moisture diffusion and drying rate in vacuum freeze-drying through microstructural, membrane, pectin, and hydrophilicity changes and improved several bioactive and physicochemical attributes.	Direct	[190]
Yardlong bean pods	Vacuum steam-pulse blanching followed by gliding-arc cold plasma	Hot-air impingement drying	Drying kinetics; Microstructure; Product quality; Biochemical/structural response	A short plasma exposure after vacuum steam-pulse blanching accelerated drying and retained folate, whereas longer exposure caused structural and oxidative damage.	Hybrid	[191]
Mussel meat	Air and argon cold plasma	Heat-pump drying	Drying kinetics; Moisture transfer/water state; Product quality	Air and argon plasma both accelerated mussel heat-pump drying; air plasma produced stronger etching and penetration, improved rehydration, and reduced lipid oxidation.	Direct	[192]
Chinese yam starch	Cold plasma	Hot-air and infrared drying	Product quality; Biochemical/structural response	CP cracked and depolymerized yam starch, reducing molecular weight and crystallinity and thereby altering solubility, swelling, pasting, digestion, and functional properties after drying.	Direct	[193]
Red beet	Cold plasma4	Alternating infrared-hot-air drying	Drying kinetics; Microstructure; Product quality; Modeling/process prediction	CP produced numerical improvements in drying time, shrinkage, and rehydration, but most effects were not statistically significant; infrared power and pulse ratio dominated the drying response.	Direct	[194]
Blueberry	Radio-frequency cold plasma	Hot-air drying	Drying kinetics; Moisture transfer/water state; Product quality	RF cold plasma gave the shortest blueberry drying time, highest diffusivity, best color and rehydration, and greatest anthocyanin and phenolic retention among the compared pretreatments.	Direct	[195]
Tomato slices	Plasma-functionalized water, alone or combined with osmotic dehydration	Hot-air drying	Drying kinetics; Moisture transfer/water state; Microstructure; Product quality	Plasma-functionalized water shortened drying and improved several quality attributes; combining it with osmotic dehydration mitigated some disadvantages of osmotic dehydration alone but reduced soluble solids.	Indirect/Hybrid	[196]
Ginger slices	Cold plasma followed by ultrasound-assisted convective drying	Ultrasound-assisted convective drying	Drying kinetics; Energy/environmental performance; Modeling/process prediction	A shorter CP exposure with lower ultrasound power slightly increased drying time relative to the high-power ultrasound control but markedly reduced exergy destruction and environmental impact.	Hybrid	[197]
White radish	Cold plasma	Hot-air drying	Drying kinetics; Moisture transfer/water state; Microstructure; Product quality	CP shortened white-radish drying by altering surface and cellular structure and improved rehydration, phenolic, ascorbic-acid, and antioxidant retention.	Direct	[198]
Apple slices	Cold plasma	Hot-air, microwave, and vacuum-microwave drying	Drying kinetics; Moisture transfer/water state; Microstructure; Product quality; Biochemical/structural response	CP shortened hot-air and vacuum-microwave drying, preserved color and hardness during storage, and shifted PPO, POD, and PAL activities toward lower browning and better phenolic retention.	Direct	[199]
Potato tissue	Nonthermal pulsed filamentary microplasma assisted by thermionic emission	Microplasma-assisted drying	Drying kinetics; Modeling/process prediction	Microplasma treatment shortened drying, and a coupled heat-mass-pressure model reproduced the experimental behavior with a reported discrepancy below 3%.	Direct	[200]
Green peas	Cold plasma	Hot-air drying	Drying kinetics; Moisture transfer/water state; Microstructure; Product quality	CP increased moisture diffusivity, shortened drying, and improved rehydration, color, phenolics, and antioxidant activity through pore formation and surface roughening.	Direct	[201]
Tomato slices	Plasma-functionalized water	Infrared-assisted pulsed-vacuum drying	Drying kinetics; Microstructure; Energy/environmental performance; Product quality	Plasma-functionalized water disrupted tomato tissue, shortened infrared-pulsed-vacuum drying, and improved rehydration, porosity, bioactives, and appearance while reducing hardness-related attributes.	Indirect	[202]
Grape	Cold plasma	Hot-air impingement drying	Drying kinetics; Microstructure; Product quality; Biochemical/structural response	CP progressively modified membrane and pectin structures and shortened drying, but prolonged treatment softened tissue; a moderate exposure best balanced drying efficiency with color and bioactive retention.	Direct	[203]
Chinese yam	Cold plasma; thermal and other nonthermal pretreatment comparators	Hot-air drying	Drying kinetics; Microstructure; Biochemical/structural response; Modeling/process prediction	Among the pretreatments compared, CP provided a modest reduction in yam drying time; microwave produced the strongest moisture-transfer and structural response.	Direct	[204]

Food material	Plasma or treatment system	Drying method	Evidence reported	Primary outcome(s)	Evidence classification	Reference
Cumin seeds	Cold plasma followed by ultrasound-assisted air drying	Ultrasound-assisted air drying	Drying kinetics; Moisture transfer/water state; Energy/environmental performance; Product quality; Modeling/process prediction	The study modelled CP- and ultrasound-assisted cumin drying; the wavelet neural network gave the best overall prediction of kinetic, energy, color, and rupture-force outcomes.	Hybrid	[205]
Blueberry	Conveyor-based tumbling gliding-arc plasma	Hot-air drying	Drying kinetics; Moisture transfer/water state; Microstructure; Energy/environmental performance; Product quality	The conveyor plasma system shortened blueberry drying, improved energy efficiency, reduced specific energy use and emissions, enhanced phenolics and antioxidant capacity, and shortened the estimated payback period.	Direct	[206]
Garlic slices	Cold plasma; ultrasound and ozone-water comparators	Hot-air drying	Drying kinetics; Microstructure; Product quality	Cold plasma was one of three separately tested pretreatments; ultrasound most improved drying kinetics, whereas ozone water produced the best nutritional-quality response.	Direct	[207]
Canola seeds	Cold plasma	Fluidized-bed air drying	Drying kinetics; Modeling/process prediction	The effect of CP depended on temperature and exposure: it shortened drying at selected 40-50 degrees C conditions but did not produce the shortest time at 60 degrees C.	Direct	[208]
Sweet lime peel	Cold plasma	Hot-air drying	Drying kinetics; Moisture transfer/water state; Microstructure; Energy/environmental performance; Product quality; Modeling/process prediction	CP shortened sweet-lime-peel drying and improved rehydration, phenolics, flavonoids, antioxidant activity, and ascorbic acid; an ANN outperformed conventional drying models.	Direct	[209]
Jujube slices	Cold plasma; HHP, ultrasound, HPCD, and blanching comparators	Vacuum freeze-drying	Microstructure; Energy/environmental performance; Product quality	The pretreatments generally lowered water activity and increased rehydration, but CP was not the best-performing treatment for overall quality; HPCD and ultrasound retained more attributes.	Direct	[210]
Jujube slices/powder	Cold plasma combined with enzyme treatment and ultrasound	Vacuum-microwave drying	Microstructure; Product quality; Biochemical/structural response	The combined CP-ultrasound-enzyme treatment most reduced sugars, hygroscopicity, and agglomeration and produced the highest measured bioactive and antioxidant indices in jujube powder.	Hybrid	[211]
Potato	Cold filamentary microplasma assisted by thermionic emission	Convective drying	Drying kinetics; Energy/environmental performance; Biochemical/structural response	Cold filamentary microplasma increased potato drying rate and shortened drying time with only minor changes in sugars, starch, and FTIR-detectable functional groups.	Direct	[212]
Winter jujube slices	Cold plasma; ultrasound comparator	Vacuum freeze-, hot-air, and microwave-coupled pulsed-vacuum drying	Product quality	CP and ultrasound were compared across three drying methods; the CP-microwave-pulsed-vacuum combination increased flavonoids and was associated with improved product quality.	Direct	[213]
Black wheat flour	Radiofrequency or cold-plasma drying	Radiofrequency or cold-plasma drying	Microstructure; Product quality; Biochemical/structural response	RF and CP altered starch and matrix structure and increased phenolic extractability; prolonged treatment weakened rheology, while CP-15 produced the best 3D-printing performance.	Direct	[214]
Apple slices	Low-temperature atmospheric plasma	Pretreatment before drying (subsequent drying method not evaluated in this study)	Product quality	Treatment duration did not significantly change moisture content. Phenolic profiles and antioxidant activity responded non-monotonically: antioxidant activity initially increased, whereas prolonged exposure reduced it below the raw-apple value; 30 min reduced browning and flavan-3-ols while slightly increasing dihydrochalcones and flavonols.	Direct	[215]

Direct: the food material was exposed directly to the plasma or discharge region. Indirect: treatment was delivered through a plasma-generated medium, such as plasma-processed air, plasma-activated water, or plasma-functionalized water. Hybrid: the plasma-related step was combined with an additional independent treatment. A plasma pretreatment followed only by the drying operation was not classified as hybrid.
